# Force-based reading and writing of individual single-atom magnets

**DOI:** 10.1038/s41467-026-74922-z

**Published:** 2026-07-09

**Authors:** Yuuki Adachi, Kazuki Ueda, Yuuki Yasui, Yoshiaki Sugimoto

**Affiliations:** Department of Advanced Materials Science, The University of Tokyo, Kashiwa, Japan

**Keywords:** Scanning probe microscopy, Imaging techniques, Surfaces, interfaces and thin films, Characterization and analytical techniques

## Abstract

The integration of single-atom bits enables the realization of the highest data-density memory. Reading and writing information to these bits through mechanical interactions opens the possibility of operating the magnetic devices with low heat generation and high density recording. To achieve this visionary goal, we demonstrate the use of magnetic exchange force microscopy to read and write the spin orientation of individual holmium adatoms on MgO thin films. The spin orientation of the holmium adatom is stabilized by the strong uniaxial anisotropy of the adsorption site and can be read out by measuring the exchange forces between the magnetic tip and the atom. The spin orientation can be written by approaching the tip closer to the holmium adatom. We explain this writing mechanism by the symmetry reduction of the adsorption site of the Ho adatom. These findings demonstrate the potential for information storage with minimal energy loss and pave the way for a new field of atomic-scale mechano-spintronics.

## Abstract (web summary)

The advance of computing has seen the progressive size reduction of magnetic memory components; the extreme limit of this miniaturization is to exploit the magnetization of a single atom. Here, Adachi and coauthors use magnetic exchange force microscopy to read and write the magnetization of a single holmium atom on a surface.

## Introduction

A hard disk drive, which is a representative nonvolatile memory device, is composed of two-state magnetic bits that store information^1,2^. Traditionally, reading information from these magnetic bits has been performed through magnetoresistive effects driven by electric currents, while writing information to these magnetic bits relies on electromagnetic induction generated by current-driven magnetic fields. However, the use of electric currents inevitably causes Joule heating^3^. Such Joule heating becomes a critical issue in high-density atomic memory arrays, where the cumulative heat can be significant. In this study, we develop current-free methods for reading and writing information to the single-atom bits, thereby achieving low heat generation and high recording density. Specifically, we propose a method for reading and writing information to the single-atom bits using force. Since our proposed method enables the reading and writing of single-atom bits by force, it can be applied to various surfaces, including bulk insulating substrates that are inaccessible to conventional electrical techniques^4^.

## Results

**Fig. 1:**
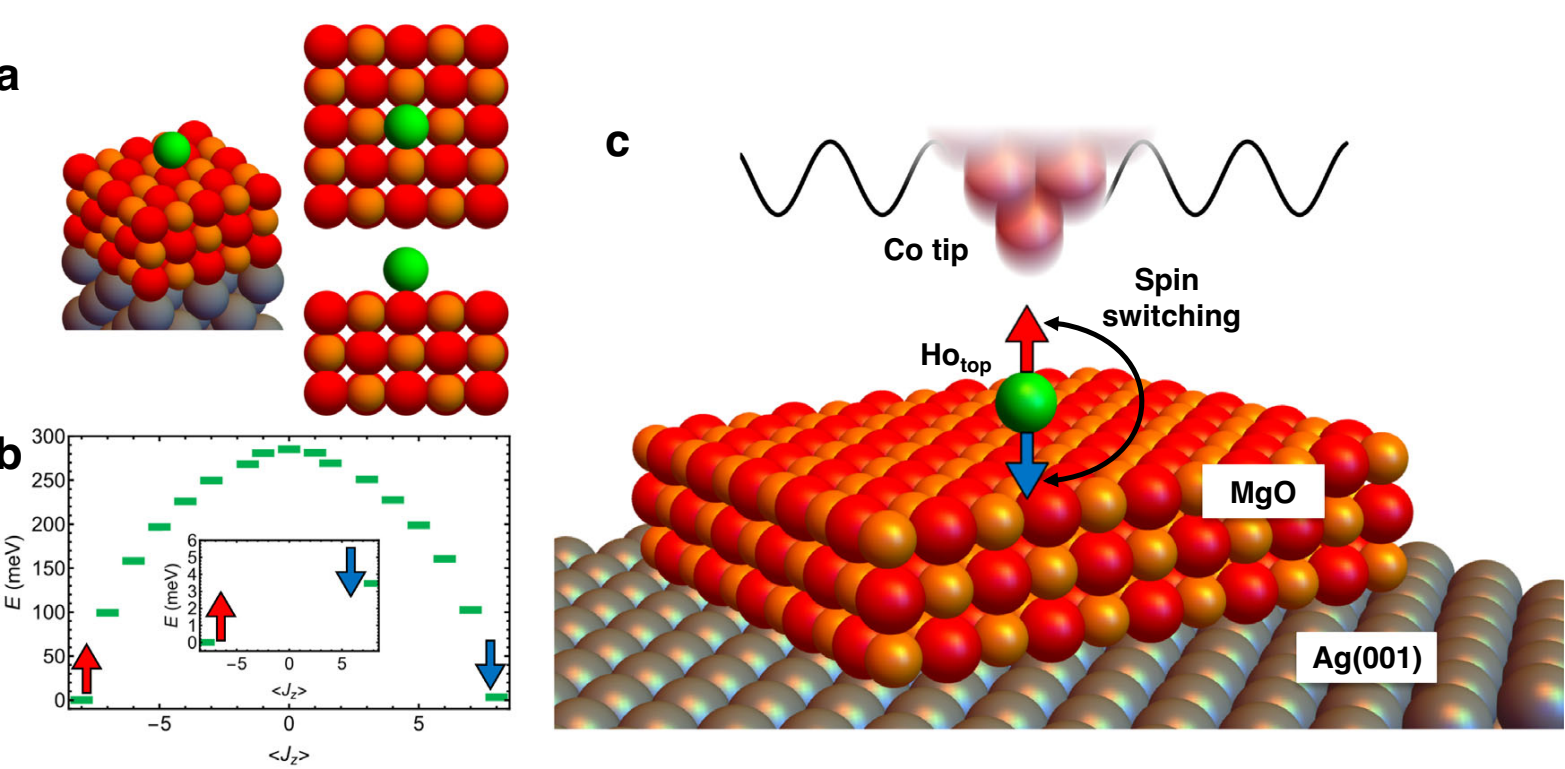
Energy diagram of holmium adatom on MgO and experimental set-up to read and write the magnetic states of Ho adatoms. **a** Three-dimensional views of the adsorption configuration of a Ho adatom at the top site in the high-symmetry *C*_4*v*_ position on MgO/Ag(100), together with top and side views of the same configuration on MgO. Green ball: Ho atom, orange ball: Mg atom, red ball: O atom, gray ball: Ag atom. **b** Calculated eigenvalues of top-site Ho in high-symmetry *C*_4*v*_ on MgO/Ag(100) at *B* = 3.0T. The large uniaxial crystal field, with only minor transverse components, suppresses efficient direct transitions between the ground and metastable states (Ho*↑* and Ho*↓*). The red and blue arrows in (**b**) indicate the Ho*↑* and Ho*↓*. The inset shows a magnified view of the low-energy region. **c** Schematic of the force-based reading and writing of single atom magnets.

Herein, we utilize holmium (Ho) adatoms on MgO as single-atom magnet, a system that is regarded as a benchmark for single-atom memory^5–12^ (see also Figs. S1 and S2 in the supporting information). A Ho adatom adsorbed on the Oxygen top site (Ho_top_) has been experimentally characterized using X-ray absorption spectroscopy (XAS), X-ray magnetic circular dichroism (XMCD) and spin-polarized scanning tunneling microscopy (SP-STM)^10,11,13^. As shown in Fig. 1(a,b), Ho_top_ has a ligand field with *C*_4*v*_ symmetry, which effectively suppresses direct transitions between the ground state and the metastable state (Ho*↑* and Ho*↓*) due to the strong uniaxial anisotropy, and thus gives rise to a long-lived magnetic quantum state with two configurations, Ho*↑* and Ho*↓* (see also Section 3 in the Supporting Information)^4,6,10,12,14^. In Fig. 1(b), under an external magnetic field of 3.0 T, the Ho state aligned with the magnetic field has an energy preference of approximately 3.5meV over the opposite state. Notably, spin switching at zero magnetic field is prevented by the hyperfine interaction^4^. These properties make Ho_top_ adatoms promising candidates for the smallest stable magnetic bits. In previous SP-STM experiments, applying a bias voltage above  ~ 100 mV allowed tunneling current to induce switching between Ho*↑* and Ho*↓* by overcoming the energy barrier ^6,10^. Compared to Ho_top_, Ho_bridge_ is located in a crystal field with *C*_2*v*_ symmetry, which represents a lower-symmetry environment (see Fig. S3). As depicted in Fig. S3b, this reduced symmetry leads to strongly mixed quantum states even under an applied magnetic field of 3.0 T, thereby shortening the magnetic lifetime^12^.

To read and write the spin orientation of a Ho adatom adsorbed on an MgO surface, we employed magnetic exchange force microscopy (MExFM)^15–20^ using a length extension resonator (LER) operated in the frequency modulation mode. In this mode, the frequency shift (Δ*f*) allows the determination of the force between the tip and the sample from the attractive regime to the repulsive regime (see also the Supporting Information)^21,22^. Fig. 1c illustrates the experimental setup. A tungsten tip functionalized with cobalt (Co) atoms at its apex was mounted on a LER and oscillated at its resonance frequency *f*_0_ ~ 1 MHz with an amplitude of *A* = 65pm. It was then used to probe a Ho adatom under near-zero bias voltage at 4.5K under an external magnetic field of 3.0T (see Figs. S4 and S5 in the Supporting Information for the preparation of the Co tip). The magnetization of the Co tip aligns with an external magnetic field due to the superparamagnetic nature of the Co cluster^10,23,24^.

**Fig. 2:**
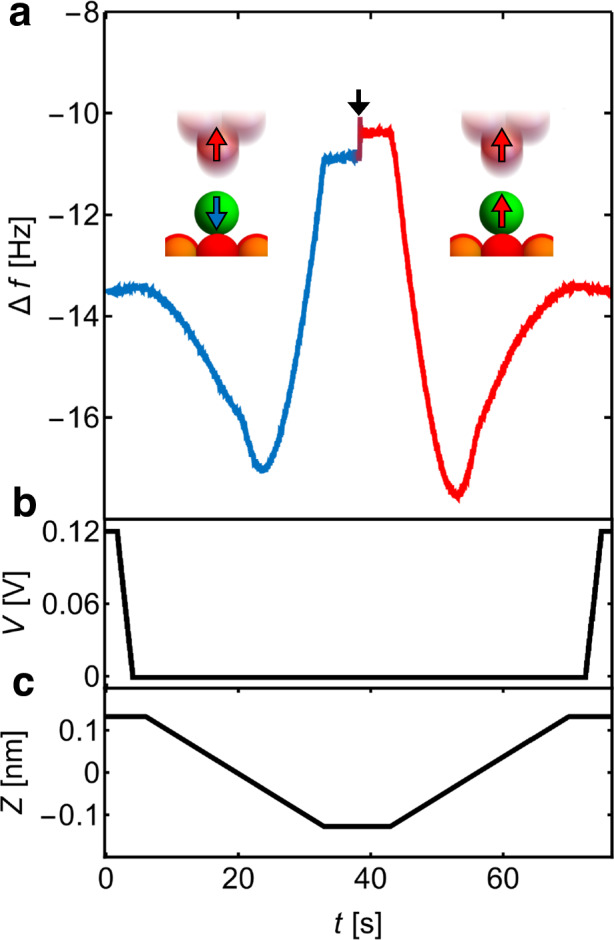
Reading and writing of Ho spin on MgO using MExFM. **a**–**c** Δ*f*(*t*) spectra measured on top of Ho_top_ while varying the bias voltage and the tip height. **a** Time evolution of Δ*f*, **b** applied bias voltage, and **c** tip-sample distance. The blue and red in (**a**) indicate the Ho*↓* and Ho*↑*. At 33s ≤*t*≤ 43s, the transition from the Ho*↓* to Ho*↑* state can be detected by a sudden jump in Δ*f*(*t*), marked by the black arrow in (**a**).

Here, we present the experimental results of force-based reading and writing Ho*↓* and Ho*↑*. Figure 2a shows a typical Δ*f* as a function of time, measured on top of Ho_top_ while varying the bias voltage (Fig. 2b) and the tip–sample distances (Fig. 2c). First, the lateral position of the tip was fixed above the center of Ho_top_. At *V* = 120 mV, the Ho spin undergoes current-induced switching. The spin state was then stabilized in the desired configuration (in this case, Ho*↓*) by lowering the bias voltage from *V* = 120 mV to *V* = 200 *μ*V, well below the threshold for current-induced spin switching (0s ≤*t*≤ 4s). Once set, the Ho*↓* state was measured via Δ*f* during the tip approach (6s ≤*t*≤ 33s). To switch the spin state from Ho*↓* to Ho*↑*, the tip was brought to a specific distance (*z* =  − 0.13 nm), exceeding the threshold distance required to induce spin switching (*z* = 0.00 nm is the point-contact distance, see also Methods). The spin state of the Ho_top_ was then probed at this distance with a fixed probe time of 10 s (33s ≤*t*≤ 43s), and the transition from Ho*↓* to Ho*↑* was detected as a sudden jump in Δ*f*, indicated by the black arrow in Fig. 2a. Afterward, the Ho*↑* was measured from Δ*f* by retracting the tip to its original tip–sample distances (43s ≤*t*≤ 70s). Finally, the bias voltage was restored from *V* = 200*μ*V to its original value of *V* = 120 mV (72s ≤*t*≤ 76s; see also Fig. S6 for the tunneling current simultaneously recorded with Δ*f* in Fig. 2a). In Fig. 2a, the minimum of Δ*f* obtained on top of Ho*↑* is smaller than that for Ho*↓*, demonstrating that the two spin states can be successfully read out by MExFM, and that the Ho spin can be written from Ho*↓* to Ho*↑* by approaching the tip.

**Fig. 3:**
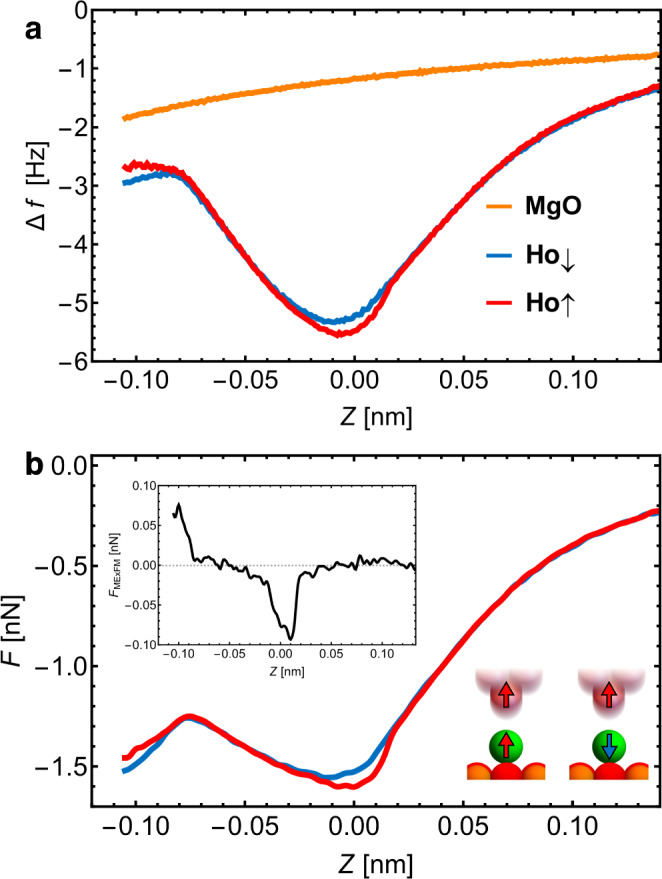
Probing Ho*↑* and Ho*↓* using MExFM. **a** Frequency shift obtained on top of the Ho*↑* (Δ*f*_Ho_*↑*(*z*), red solid curve), Ho*↓* (Δ*f*_Ho_*↓*(*z*), blue solid curve) and MgO (Δ*f*_MgO_(*z*), orange solid curve). Measurement conditions: *V* = 200*μ*V. **b** Short-range forces obtained on top of Ho*↑* (*F*_Ho_*↑*(*z*), red solid curve) and Ho*↓* (*F*_Ho_*↓*(*z*), blue solid curve). Inset in (**b**) shows magnetic exchange force *F*_MExFM_(*z*) obtained on top of the Ho adatom. The gray dotted line is a guide for the eye, indicating *F*_MExFM_(*z*) = 0.

To discuss the reading mechanism of Ho*↑* and Ho*↓*, here we show the short-range force and magnetic exchange force recorded on top of Ho_top_ (see Figs. S7 and S8 for full data sets and dissipation). Figure 3a shows the Δ*f* as a function of tip–sample distance (Δ*f*(*z*)), recorded on top of Ho*↑*, Ho*↓* and MgO (Δ*f*_Ho↑_(*z*), Δ*f*_Ho↓_(*z*) and Δ*f*_MgO_(*z*)). Both Δ*f*_Ho↑_(*z*) and Δ*f*_Ho↓_(*z*) include a long-range component from the MgO substrate. Therefore, Δ*f*_MgO_(*z*) was subtracted from Δ*f*_Ho↑_(*z*) and Δ*f*_Ho↓_(*z*) to eliminate the background component. As shown in Fig. 3b, the short-range force on Ho*↑* and Ho*↓* (*F*_Ho↑_(*z*) and *F*_Ho↓_(*z*)) were calculated from the background subtracted Δ*f*(*z*) ^22^. In Fig. 3b, as the tip approaches the Ho*↑* (Ho*↓*) adatom, *F*_Ho↑_(*z*) (*F*_Ho↓_(*z*)) exhibits *F*_Ho↑_ (*z* = 0.00 nm)  = − 1.60 nN (*F*_Ho↓_ (*z* = 0.00 nm)  = − 1.55 nN), indicating ferromagnetic coupling between the Co tip and the Ho adatom at this distance. Reducing *z* further decreases the attractive force to *F*_Ho↑_ (*z* = − 0.08 nm)  = − 1.25 nN (*F*_Ho↓_ (*z* = − 0.08 nm)  = − 1.25 nN). As *z* is reduced even more, the attraction increases, reaching *F*_Ho↑_ (*z* = − 0.10 nm)  = − 1.40 nN (*F*_Ho↓_ (*z* = − 0.10 nm)  = − 1.50 nN), indicating antiferromagnetic coupling at this distance. The inset in Fig. 3b shows the magnetic exchange force, *F*_MExFM_(*z*), derived by subtracting *F*_Ho↓_(*z*) from *F*_Ho↑_(*z*). As the tip approaches, a transition from ferromagnetic to antiferromagnetic coupling can be observed (ferromagnetic: *F*_MExFM_(*z*) < 0 and antiferromagnetic: *F*_MExFM_(*z*) > 0).

The ferromagnetic coupling between the highly localized 4*f* electrons in the Ho adatom and the 3*d* electrons in the Co tip can be explained by two contributions: first, an intra-atomic ferromagnetic coupling between the 4*f* and 5*d* (or 6*s*) spins within the Ho adatom, and second, an inter-atomic ferromagnetic coupling between the 5*d* (or 6*s*) electrons of Ho and the 3*d* electrons of the Co atom^10,25,26^. The transition from ferromagnetic to antiferromagnetic coupling is reported for the interaction between the 5*d* electrons of Ta and the 3*d* electrons of Fe^27^. The transition from ferromagnetic to antiferromagnetic coupling is reminiscent of the Bethe-Slater curve. The antiferromagnetic coupling between the highly localized 4*f* electrons in the Ho adatom and the 3*d* electrons in the Co tip can be explained by an intra-atomic ferromagnetic coupling between the 4*f* and 5*d* (or 6*s*) electrons within the Ho adatom, and an inter-atomic antiferromagnetic coupling between the 5*d* (or 6*s*) electrons of Ho and the 3*d* electrons of the Co atom.

**Fig. 4:**
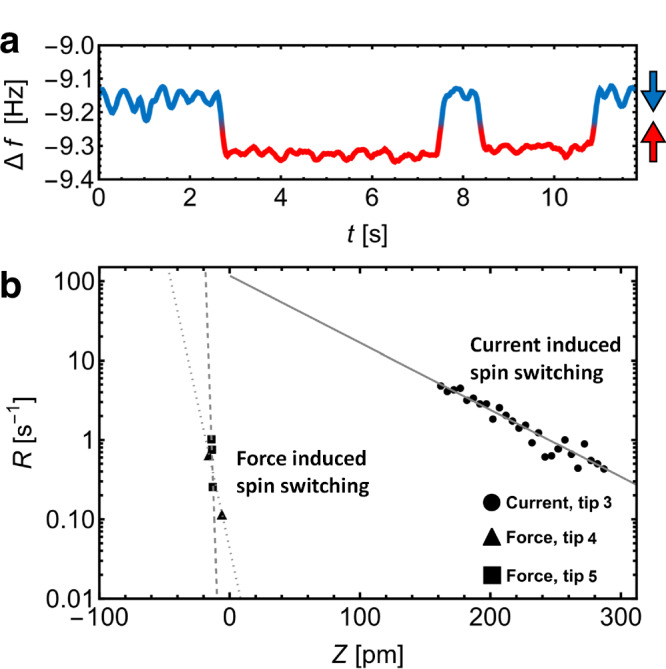
Switching between Ho*↑* and Ho*↓.* **a** Telegraph signal due to the force-induced magnetic switching between Ho*↑* and Ho*↓*. The blue and red indicate the Ho*↓* and Ho*↑*. Measurement conditions: constant-height mode, *V* = 1.0 mV, *z* = −16.0 pm. **b** Spin switching rate as a function of tip-sample distances. The exponential fits are represented by the solid, dashed and dotted lines. Measurement conditions: constant height mode, *V* = 1.0 mV for force induced spin switching and *V* = 150 mV for current induced spin switching.

As we discussed in Figs. 2(a–c), the Ho spin can be switched from Ho*↓* to Ho*↑* by approaching the tip. In Fig. 4(a), we further demonstrate bidirectional switching between Ho*↓* and Ho*↑* induced by the tip approach. Firstly, the tip was brought above the center of Ho_top_, and the bias voltage was set to *V* = 200*μ*V to avoid spin switching induced by the tunneling current. Then, the tip-sample distance was adjusted to values exceeding the threshold distance required to induce spin switching via tip approach. As shown in Fig. 4(a), the spin switching was monitored in real time by recording the Δ*f* while keeping the tip height constant. Telegraph noise between the two states was observed, indicating bidirectional spin switching between Ho*↑* and Ho*↓*. Therefore, due to the bidirectional spin switching, we can control the spin not only from Ho*↓* to Ho*↑* (as demonstrated in Figs. 2(a–c)) but also from Ho*↑* to Ho*↓*, as shown in Fig. S9. Moreover, the observation of the bidirectional spin switching rules out exchange forces as the driving mechanism for spin switching^28^. Fig. 4b summarizes the switching rates between Ho*↑* and Ho*↓* with the results of spin switching induced by the tunneling current (see also Fig. S10). In Fig. 4b, the spin switching induced by tip approach decays more rapidly along with distance than that induced by the tunneling current.

To discuss the writing mechanism of Ho*↑* and Ho*↓*, the tip was positioned at the center of Ho_top_ and approached closer than the spin-switching distance. As shown in Fig. S11, this approach induced a lateral displacement of Ho_top_ to the bridge site, resulting in the formation of Ho_bridge_. These results demonstrate that lateral displacement from Ho_top_ to Ho_bridge_ can be induced even when the tip approaches the center of Ho_top_ at a specific tip-sample distance. Because the Co tip has an asymmetric shape (see Fig. S12(a–d)), multiple Co atoms are expected to come into contact with the Ho adatom during relaxation, thereby inducing lateral displacement. Notably, once the Ho adatom relocates to the Ho_bridge_, it rarely returns to Ho_top_.

The lateral displacement from Ho_top_ to Ho_bridge_, in turn, strongly influences the spin state of the Ho adatom. Specifically, as shown in Figs. 1b and S3b, transitions between Ho*↑* and Ho*↓* are suppressed for Ho_top_, whereas the reduced-symmetry Ho_bridge_ exhibits strongly mixed quantum states, enabling direct transitions between Ho*↑* and Ho*↓* even under an applied magnetic field of 3.0 T (see also Section 3 in the Supporting Information)^12^. The importance of crystal-field symmetry for magnetic stability has been widely reported in other systems^8,29–31^. We therefore propose that spin-switching of a single atom is driven by strain-induced state mixing using an atomic probe. The spin switching between Ho*↑* and Ho*↓* occurs when Ho_top_ moves toward Ho_bridge_ but does not fully reach it, due to the force exerted by the Co tip. This is further confirmed as in Fig. S13, which shows that the spin switching distance varies depending on the tip shape, but spin switching always occurs at distances shorter than the point-contact distance. Although the spin switching distance depends on the tip shape, once an appropriate tip is prepared, the spin orientation and switching rate can be controlled by adjusting the tip-sample distance, as demonstrated in Figs. 2, S9, and 4(a,b), thereby enabling controlled writing of a single-atom magnet using force.

**Fig. 5:**
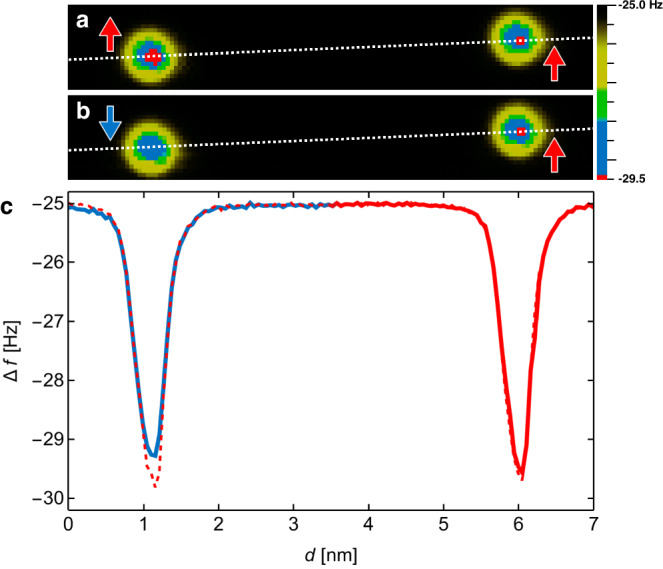
Imaging Ho*↑* and Ho*↓* using MExFM. **a** Δ*f* image of two Ho adatoms, both Ho adatom in the Ho*↑*. Imaging parameters: constant height mode, *V* = 200*μ*V, scan size 1.2 nm  × 7.0 nm. **b** Δ*f* image of the same area in (**a**), after the left Ho adatom was manipulated from Ho*↑* to Ho*↓*. Imaging parameters: constant height mode, *V* = 200*μ*V, scan size 1.2 nm  × 7.0 nm. **a** and **b** were obtained at the same tip height. **c** Line profiles obtained above the Ho adatoms by the dotted curve for (**a**) and the solid curve for (**b**). The blue and red indicate the Ho*↓* and Ho*↑*. The positions of the line profiles are indicated by the dotted lines in (**a**) and (**b**).

Beyond merely reading Ho*↓* and Ho*↑* through its spectroscopy capabilities, MExFM enables imaging of Ho*↓* and Ho*↑*. As shown in Figs. 5(a,b), this is achieved by scanning the tip horizontally at a constant height while recording Δ*f*. The tip-sample distance is set to be approximately 20 pm larger than the point-contact distance to avoid spin switching. In Fig. 5a, both Ho adatoms appear in the Ho*↑* state. To demonstrate the spin-readout capability, we switched the spin state of the left Ho adatom from Ho*↑* to Ho*↓* and imaged the same area again using the same tip in Fig. 5a. In Fig. 5b, the left Ho adatom appears in the Ho*↓* state, whereas the right one remains in the Ho*↑* state. In Fig. 5c, the contrast changed only for the spin switched Ho atom, while the unswitched reference Ho atom remained identical. This observation allows us to rule out the possibility of a tip change during the Ho spin manipulation. In Figs. 5a and 5b, based on the time required to image a single Ho, the Ho spin can be stably read out for at least 218s. Consequently, our force-based approach remains intrinsically non-invasive during readout. Therefore, we successfully demonstrated the readout of Ho adatom spin orientations in both configurations using MExFM.

In this work, we show that the spin orientation of a single-atom magnet can be read and written using force, specifically by means of MExFM. We demonstrate this by probing individual Ho adatoms on MgO thin films, distinguishing between the Ho*↑* and Ho*↓* states through exchange forces, and controlling these states by adjusting the tip-sample distance to induce lateral displacement and manipulate the adsorption-site symmetry. The method of controlling the spin by manipulating the adsorption-site symmetry, as proposed in this study, is not specific to our system^31^. This opens new possibilities for manipulating spin states via the surrounding atomic environment. Spin detection and manipulation of 4*f*-electron systems by a nondissipative force, unlike electric currents, is expected to lead to the realization of long spin coherence times, which are critical for quantum information processing^32–34^.

## Methods

### MExFM measurements

The experiments were conducted using a custom-built scanning probe microscope operating under ultra-high vacuum conditions at 4.5K^35^. A commercial quartz length-extension resonator (LER), known as a Kolibri sensor (SPECS) and equipped with a tungsten (W) tip, was utilized. The microscope is a combined AFM/STM system capable of applying magnetic fields perpendicular to the sample surface. The experiments were carried out with magnetic fields at 3.0T (see also sections 14 and 16 in the supporting information). For differential conductance (d*I*/d*V*) spectroscopy, we employed a lock-in technique with a modulated sample bias voltage of 2.0 mV at 617 Hz. *I*_average_ indicates the time-averaged tunneling current measured by oscillating the tip at an amplitude of 65 pm. *I* indicates the static tunneling current measured without oscillation.

### Sample preparations

The Ag(001) surface was cleaned by repeated cycles of Ar^+^ sputtering followed by annealing at 600 ^∘^C. The quality of Ag(001) was confirmed by STM imaging (Fig. S1a). After confirming the clean Ag(001), Mg was deposited in an oxygen background pressure of 2.0 × 10^−7^ torr at a rate of ~0.2 ML/min while the Ag(001) crystal was maintained at 600 ^∘^C. The temperature was then slowly decreased to room temperature over a period of ~45 min^36–40^. The quality of the MgO thin film (≥ 2 ML) was confirmed by STM imaging and field-emission measurements (Fig. S1b and S1d)^40^. All the data are obtained on 3 ML films. A high-purity (99.9%) Ho rod was cleaned by filing the oxidized surface layer until a shiny metallic surface was exposed. To minimize exposure to ambient conditions, the Ho rod was immediately transferred into the ultra-high vacuum chamber. Both holmium and cobalt atoms were deposited onto the MgO/Ag(001) at 4.5K (Fig. S1c).

*z* = 0 is defined as the tip–atom distance, measured from the point conductance of Ho adatom^21,41^. When a Co tip was used, the point of conductance of Ho*↓* was taken as the reference. We used point conductance *G*_0_ = 7.748 × 10^−5^ S.

### The software used to create the figures

Figure 1 c, Supplementary Fig. 2(d-f), and Supplementary Fig. 3a were generated using Mathematica, while Supplementary Fig. 8(b,c) was generated using Microsoft PowerPoint.

## Supplementary information

Supplementary information

Peer Review File

## Data Availability

The data that support the findings of this study are available from the corresponding author upon request.
