# Supplementary material for: Force-based reading and writing of individual single-atom magnets
Source: Nat Commun. 2026 Jul 9;17:5927. doi: 10.1038/s41467-026-74922-z (PMC13350084; doi:10.1038/s41467-026-74922-z)
Supplement: Supplementary file 1 — Supplementary information [file 41467_2026_74922_MOESM1_ESM.pdf]

Supporting information: Force-Based Reading and Writing of Individual Single-Atom Magnets

Yuuki Adachi[1], Kazuki Ueda[1], Yuuki Yasui[1], Yoshiaki Sugimoto[1*]

[1]Department of Advanced Materials Science, The University of Tokyo, Kashiwa, Chiba 277-8561, Japan.

*Corresponding author E-mail: ysugimoto@k.u-tokyo.ac.jp

1 Summary of the Co tips used for each figure

We have used several different Co tips for spin switching. To make it easier to distinguish them, we assigned the same numerical label to the same tip, as shown in Supplementary Table 1.

Supplementary Table 1 Summary of the tips used for each figure in the main text and supplementary information. The same numerical label indicates the same tip.

Figure	Tip Used
Fig. 2	Tip 1
Fig. 3	Tip 2
Fig. 4	Tips (3–5)
Fig. 5	Tip 6
Fig. S1	Tip 7
Fig. S2	Tip 8
Fig. S4	Tip 9
Fig. S5	Tip 10
Fig. S6	Tip 1
Fig. S7	Tip 2
Fig. S8	Tip 11
Fig. S9	Tip 12
Fig. S10	Tip 3
Fig. S11	Tip 13
Fig. S12	Tip 14
Fig. S13	Tip 1, Tip 4, Tip 12 and Tips (15–21)
Fig. S14	Tip 22
Fig. S15	Tip 23

2 Observation of coadsorbed Ho and Co atoms on MgO/Ag(001)

Fig. S2a shows an STM image of coadsorbed Ho and Co atoms on MgO/Ag(001). Different types of stable bright features are observed on top of the MgO surface. Figs. S2b and S2c show line profiles and dI/dV spectra measured on top of them. $\mathrm{Ho_{top}}$ and $\mathrm{Ho_{bridge}}$ are distinguished by their different apparent heights (Fig. S2b). Moreover, in both cases, no inelastic conductance steps were observed (Fig. S2c) [1]. Co adatoms are identified by their dI/dV steps at 58 meV, reflecting their high magnetic anisotropy (Fig. S2c) [1, 2]. Schematics of these adatoms are shown in Figs. S2(d—f).

3 The effective spin Hamiltonian analysis

We model the energy diagram of Ho adatom using an effective spin Hamiltonian of the form:

$$\hat{H} = \hat{H}_{\mathrm{cf}} + \hat{H}_{\mathrm{Z}} \tag{S1}$$

with H_{cf} describing the effects of the crystal field, and H_{Z} describing the effects of the external magnetic field. We use this effective spin Hamiltonian to describe the splitting of the lowest multiplet of magnetic states. For the Ho adatom in the $4f^{10}$ configuration, the lowest multiplet consists of states with a total magnetic moment $J = 8$ [3, 4]. This quantum number determines the corresponding multiplicity of the states, $2J + 1 = 17$.

For the Ho atoms adsorbed on the top site ($\mathrm{Ho_{top}}$), we include the Stevens operators permitted by the four-fold symmetry:

$$\hat{H}_{\mathrm{cf}\,C_{4v}} = B_2^0\hat{O}_2^0 + B_4^0\hat{O}_4^0 + B_4^4\hat{O}_4^4 + B_6^0\hat{O}_6^0 + B_6^4\hat{O}_6^4 \tag{S2}$$

On the other hand, for the Ho atoms adsorbed on the bridge site ($\mathrm{Ho_{bridge}}$), we include the Stevens operators permitted by the two-fold symmetry:

$$\hat{H}_{\mathrm{cf}\,C_{2v}} = B_2^0\hat{O}_2^0 + B_4^0\hat{O}_4^0 + B_6^0\hat{O}_6^0 + B_2^2\hat{O}_2^2 + B_4^2\hat{O}_4^2 + B_4^4\hat{O}_4^4 + B_6^2\hat{O}_6^2 + B_6^4\hat{O}_6^4 + B_6^6\hat{O}_6^6 \tag{S3}$$

The values of the B_n^k coefficients determine the zero-field splitting of the magnetic states. $\hat{O}_n^k$ is the Stevens operators. To calculate B_n^k, we employ the Stevens operator equivalent method [5]. B_n^k can then be written as:

$$B_n^k = -\frac{Q_{\mathrm{M}}}{4\pi\epsilon_0}\sum_{l=1}^{L}\frac{4\pi}{(2n+1)}\frac{(-1)^k Y_n^{-k}(\theta_l,\phi_l)}{R_l^{(n+1)}} q_l \langle r^n\rangle y_n^k \theta_n \tag{S4}$$

where Q_{M} is the total charge of a Ho adatom, ε_0 is the dielectric constant, Y_n^k is the spherical harmonics, R_l is the distance between the Ho adatom and the surrounding ligand atoms and q_l is the charge on the ligand atom, the sum is over all L ligand atoms. $\langle r^n\rangle$ can be derived from the references for the Ln ions [6]. y_n^k is the numerical

coefficient occurring in Y_n^k, θ_n is the multiplicative factor [7]. We used the crystal field coordination and the charge shown in Supplementary Table 2 and Supplementary Table 3 [8]. Supplementary Table 4 summarizes the resulting coeffcients of the Stevens operators for $\mathrm{Ho_{top}}$ and $\mathrm{Ho_{bridge}}$ obtained from Eq. (S4). In Supplementary Table 4, $\mathrm{Ho_{top}}$ exhibits a large uniaxial anisotropy term ($B_2^0 = -1387$ μeV), while $\mathrm{Ho_{bridge}}$ shows a large transverse anisotropy term ($B_2^2 = 1323$ μeV).

Supplementary Table 2 Vertical distances ($d_\perp$) and lateral distances ($d_\parallel$) (in pm) between the $\mathrm{Ho_{top}}$ and the surrounding ions [8].

Ion	Charge	$d_\perp$ (pm)	$d_\parallel$ (pm)
O (underneath)	$-2e$	213	0
Mg	$+2e$	276	213
O	$-2e$	275	294

Supplementary Table 3 Vertical distances ($d_\perp$) and lateral distances ($d_\parallel$) (in pm) between the $\mathrm{Ho_{bridge}}$ and the surrounding ions [8].

Ion	Charge	$d_\perp$ (pm)	$d_\parallel$ (pm)
Mg	$+2e$	248	157
O	$-2e$	173	139

Supplementary Table 4 Calculated crystal field parameters used in effective spin Hamiltonian for $\mathrm{Ho_{top}}$ and $\mathrm{Ho_{bridge}}$. Here we used $Q_\mathrm{M} = +3e$ [9].

	$\mathrm{Ho_{top}}$	$\mathrm{Ho_{bridge}}$
B_2^0	-1387 μeV	-563 μeV
B_4^0	-831 neV	359 neV
B_6^0	-3.67 neV	1.56 neV
B_2^2		1323 μeV
B_4^2		2.49 μeV
B_4^4	231 neV	-533 neV
B_6^2		9.31 neV
B_6^4	1.53 neV	11.5 neV
B_6^6		1.20 neV

The Zeeman term in the Hamiltonian describes the interaction between the Ho spins and the external magnetic field. It reads:

$$\hat{H}_{\mathrm{Z}} = g_{\mathrm{eff}} \hat{J}_{\mathrm{z}} B \mu_{\mathrm{B}} \tag{S5}$$

In this study, we consider the ground state with $J_z = 8$ and $g_{\mathrm{eff}} = 1.25$ [4]. Supplementary Table 5 shows the eigenstates of the ground state and metastable state (Ho↑ and Ho↓) for $\mathrm{Ho_{top}}$ and $\mathrm{Ho_{bridge}}$ ($|\psi_0\rangle$ and $|\psi_1\rangle$ correspond to the eigenvectors of Ho↑ and Ho↓ in Fig. 1b and Fig. S3b, respectively), obtained by diagonalizing Eq. (S1) at $B = 3.0$ T.

The coefficient of transition probability between Ho↑ and Ho↓, induced by either electron spin–spin scattering or spin–phonon scattering, can be calculated using the following expression: [8, 10, 11]:

$$I_{01} = \frac{1}{2}\left\{ \left|\langle\psi_1|\hat{J}_+|\psi_0\rangle\right|^2 + \left|\langle\psi_1|\hat{J}_-|\psi_0\rangle\right|^2 + 2\left|\langle\psi_1|\hat{J}_z|\psi_0\rangle\right|^2 \right\} \tag{S6}$$

The first two terms enable $\Delta m = \pm 1$ transitions and the third term allows $\Delta m = 0$, where Δm is the difference in the magnetic quantum number.

Fig. 1b shows the corresponding energy-level diagram for $\mathrm{Ho_{top}}$ obtained by diagonalization of Eq. (S1) at $B = 3.0$ T. We obtain ground state and metastable state (Ho↑ and Ho↓ in Fig. 1b) with $\langle J_z\rangle = -8.0$ and $\langle J_z\rangle = +8.0$. As shown in Supplementary Table 5, these states are composed of $|m\rangle = \pm|8\rangle$ states with more than 99 % weight. No $\Delta m = \pm 1$ or $\Delta m = 0$ transitions are allowed between Ho↑ and Ho↓. Thus, according to Eq. (S6), $\mathrm{Ho_{top}}$ exhibits a suppression of direct transition.

On the other hand, Fig. S3b shows the energy-level diagram for $\mathrm{Ho_{bridge}}$ obtained by diagonalizing Eq.(S1) at $B = 3.0$ T. The ground state and metastable state (Ho↑ and Ho↓ in Fig.S3b) have $\langle J_z\rangle = -6.59$ and $\langle J_z\rangle = +6.53$. As indicated in Supplementary Table 5, these states exhibit a $\Delta m = 0$ transition with an energy difference of 3.00 meV.

The experimental results suggest that spin switching between Ho↑ and Ho↓ occurs when $\mathrm{Ho_{top}}$ moves toward $\mathrm{Ho_{bridge}}$ but does not fully reach it, due to the force exerted by the Co tip. To investigate this, we calculate the relative transition probability between the Ho↑ and Ho↓ as a function of a linear combination of $\hat{H}_{\mathrm{cf}\,C_{2v}}$ and $\hat{H}_{\mathrm{cf}\,C_{4v}}$ [3]. Specifically, we first diagonalize the Hamiltonian $H = \alpha\hat{H}_{\mathrm{cf}\,C_{2v}} + (1-\alpha)\hat{H}_{\mathrm{cf}\,C_{4v}} + \hat{H}_{\mathrm{Z}}$, and then evaluate the relative coefficient of transition probability using Eq. (S6) as a function of α. As shown in Fig. S3c, $\hat{H}_{\mathrm{cf}\,C_{4v}}$ ($\alpha = 0$) exhibits a lower transition probability than $\hat{H}_{\mathrm{cf}\,C_{2v}}$ ($\alpha = 1$), as expected. Furthermore, Fig. S3c shows that the relative transition probability changes approximately exponentially with respect to α.

Supplementary Table 5 Calculated eigenvectors for the ground state and metastable state, obtained by diagonalization of (S1) at $B = 3.0$ T.

	$\mathrm{Ho_{top}}$		$\mathrm{Ho_{bridge}}$	
$\lvert m \rangle$	$\lvert \psi_0 \rangle$	$\lvert \psi_1 \rangle$	$\lvert \psi_0 \rangle$	$\lvert \psi_1 \rangle$
$\lvert +8 \rangle$	0.00	0.9999	0.073	0.72
$\lvert +7 \rangle$	0.00	0.00	0.00	0.00
$\lvert +6 \rangle$	0.00	0.00	-0.067	-0.59
$\lvert +5 \rangle$	0.00	0.00	0.00	0.00
$\lvert +4 \rangle$	0.00	-0.0017	0.046	0.32
$\lvert +3 \rangle$	0.00	0.00	0.00	0.00
$\lvert +2 \rangle$	0.00	0.00	-0.042	-0.15
$\lvert +1 \rangle$	0.00	0.00	0.00	0.00
$\lvert 0 \rangle$	0.00	0.00	0.067	0.058
$\lvert -1 \rangle$	0.00	0.00	0.00	0.00
$\lvert -2 \rangle$	0.00	0.00	-0.14	-0.012
$\lvert -3 \rangle$	0.00	0.00	0.00	0.00
$\lvert -4 \rangle$	-0.0017	0.00	0.31	-0.024
$\lvert -5 \rangle$	0.00	0.00	0.00	0.00
$\lvert -6 \rangle$	0.00	0.00	-0.58	0.067
$\lvert -7 \rangle$	0.00	0.00	0.00	0.00
$\lvert -8 \rangle$	0.9999	0.00	0.73	-0.098
Energy (meV)	0.00	3.47	0.00	3.00

4 Fabrication of Co tip

In our experiment, the fabrication of the magnetic tip was carried out as follows [12]. First, we scanned the Co adatoms using a non-magnetic (W or Ag) tip. Fig. S4a shows the STM topography image of coadsorbed Co and $\mathrm{Ho_{top}}$ adatoms on MgO obtained by the non-magnetic tip. Next, the non-magnetic tip was positioned above the target Co adatom (highlighted by the pink arrow in Fig. S4a) and moved vertically toward the Co adatom by approximately 0.18 nm from the STM set point (Fig. S4c). After that, a bias pulse of 600 mV for 0.1 s was applied (Fig. S4c). Subsequently, we rescanned the same area and confirmed that the target Co adatom had disappeared, suggesting that Co atoms were picked up onto the tip apex (Fig. S4b). The $\mathrm{Ho_{top}}$ was then probed to observe spin switching using constant current mode ($I = 1$ nA and $V = 200$ mV), verifying the formation of the stable Co tip (see also Fig. S5b and Fig. S5e). This procedure was typically repeated several times to ensure a stable Co tip.

5 Probing Ho and Co atoms on MgO/Ag(001) using non-magnetic tip and magnetic tip

Fig. S5(a–c) shows the time dependence of the tip–sample distance recorded over $\mathrm{Ho_{top}}$, $\mathrm{Ho_{bridge}}$, and Co adatoms on MgO in constant-current mode using a non-magnetic tip, respectively. Fig. S5(d–f) presents the corresponding measurements on the same $\mathrm{Ho_{top}}$, $\mathrm{Ho_{bridge}}$, and Co adatoms as in Figs. S5(a–c), immediately after a Co adatom was picked up from the surface, as described in Fig. S4. In Figs. S5(a–f), the tunneling current and the bias voltage were set to values suitable for inducing spin

switching by current ($I = 1$ nA and $V = 200$ mV) [3, 9]. The difference in tunneling magnetoresistance between the Ho↑ and Ho↓ states leads to a change in the tip–sample distance; consequently, spin switching appears as a random telegraph signal [3, 9]. As shown in Fig. S5e, the spin switching is observed for $\mathrm{Ho_{top}}$ when using a magnetic tip.

6 Tunneling current simultaneously recorded with Δf in Fig.2a

Fig. S6a shows the averaged tunneling current recorded simultaneously with Δf in Fig. 2a. In Fig. S6a, the averaged tunneling current after restoring the bias voltage ($t = 76$ s) is larger than that before reducing the bias voltage ($t = 0$ s). These results further support the spin switching from Ho↓ to Ho↑ by approaching the tip toward $\mathrm{Ho_{top}}$.

7 Conductance and force spectroscopy measured on top of the Ho↑ and the Ho↓

Fig. S7a shows the distance dependence of Δf recorded on top of the Ho↑, Ho↓ and MgO substrate, respectively. Note that Fig. 3a shows the range -0.11 nm $\leq z \leq$ 0.14 nm of Fig. S7a. The $\Delta f(z)$ recorded over a Ho adatom includes a long-range force from the MgO substrate. Therefore, $\Delta f(z)$ measured on top of the MgO surface was subtracted from that measured over the Ho adatom to eliminate its background component. Fig. S7b shows the distance dependence of the background-subtracted short-range force. Fig. 3b shows the range -0.11 nm $\leq z \leq 0.14$ nm of Fig. S7b.

Fig. S7c shows the distance dependence of the time-averaged tunneling current, recorded simultaneously with Δf in Fig. S7a. Fig. S7d shows the distance dependence of conductance at the lowest turnaround point of the tip oscillation cycle, obtained by deconvolving the time-averaged tunneling current in Fig. S7c.

In Fig. S7b and Fig. S7d, we observe the short-range force of $F_{\mathrm{Ho}\uparrow}(z = 0.00$ nm$) = -1.60$ nN and $F_{\mathrm{Ho}\downarrow}(z = 0.00$ nm$) = -1.55$ nN, concomitant with a point-contact conductance [13, 14]. For smaller tip–sample distances, the force increases up to $F_{\mathrm{Ho}\uparrow}(z = -0.08$ nm$) = -1.25$ nN and $F_{\mathrm{Ho}\downarrow}(z = -0.08$ nm$) = -1.25$ nN, and then decreases to $F_{\mathrm{Ho}\uparrow}(z = -0.10$ nm$) = -1.40$ nN and $F_{\mathrm{Ho}\downarrow}(z = -0.10$ nm$) = -1.50$ nN, as discussed in Fig. 3. As shown in Fig. S7d, the conductance in the contact regime deviates from exponential decay, exhibiting characteristics of quantum conductance [15]. However, the applied bias voltage is limited to 200 μV. Under these conditions, spin switching for $\mathrm{Ho_{top}}$ is inhibited due to the energy barrier between the $\mathrm{Ho_{up}}$ and $\mathrm{Ho_{down}}$ as shown in Fig.1b. This suggests that the observed switching is not a result of tunneling current but involves strain-induced state mixing via the atomic probe.

Fig. S7e shows the dissipation signal as a function of tip–sample distance, simultaneously recorded with $\Delta f_{\mathrm{Ho}\uparrow}(z)$ and $\Delta f_{\mathrm{Ho}\downarrow}(z)$ in Fig. S7a. The negligible dissipation signal indicates that the position of the Ho adatom is determined for each tip–sample distance and that it is independent of the tip oscillation. In other words, when the Ho adatom moves laterally, the force between the Ho adatom and the Co tip exhibits no hysteresis throughout the tip oscillation.

8 $\Delta f(z)$ measured on top of the Ho_{top}

Fig. S8a shows the distance dependence of Δf recorded on top of the Ho_{top}. As the Co tip approaches the Ho adatom, Δf reaches a local minimum of $\Delta f = -2.7$ Hz near $z = 0$ nm. Further reduction of the distance z first leads to a slight increase and then decreases to another local minimum of $\Delta f = -5.1$ Hz at $z = -0.10$ nm.

This trend is also observed in Fig. S7a, Fig. S11b, Fig. S12a and Fig. S14g and can be explained by the lateral motion of the Ho adatom beneath the tip. Fig. S8b and Fig. S8c provide a schematic illustration of this lateral motion. When the tip–sample distance is large (Fig. S8b), the Δf curve near $z = 0$ nm reflects the interaction between the frontmost Co atom of the tip and the Ho adatom. As the tip–sample distance decreases (Fig. S8c), the Ho adatom laterally moves beneath the tip and begins to interact with Co atoms on the tip other than the frontmost atom. Similar behavior has been discussed for the tip apex atom on NaCl(100), and for the Si(001) [16, 17]. Hence, Fig. S8 supports that Ho_{top} moves laterally on MgO, due to the force exerted by the Co tip.

9 Controlling Ho spin from Ho↑ to Ho↓ by approaching the tip

In Fig. S9, employing the same approach of bringing the tip toward Ho_{top} as in Fig. 2a, we show the reverse process, namely the readout of Ho↑ and Ho↓, as well as spin switching from Ho↑ to Ho↓. Fig. S9a and Fig. S9b show typical Δf and averaged tunneling current as a function of time, measured on top of Ho_{top} while varying the bias voltage (Fig. S9c) and the tip–sample distances (Fig. S9d). As discussed in Fig.2, first, the lateral position of the tip was fixed above Ho_{top}, and its spin state was stabilized in the desired configuration (in this case, Ho↑) by lowering the bias voltage from $V = 120\,\text{mV}$ to $V = 200\,\mu\text{V}$, well below the threshold for current-induced spin switching ($0\,\text{s} \leq t \leq 3\,\text{s}$). Once set, the Ho↑ state was measured via Δf during the tip approach ($5\,\text{s} \leq t \leq 8\,\text{s}$). To switch the spin state from Ho↑ to Ho↓, the tip was brought to a specific distance ($z = -0.03$ nm), exceeding the threshold required to induce spin switching. The spin state of the Ho adatom was then probed at this distance with a fixed probe time of 10 s ($8\,\text{s} \leq t \leq 18\,\text{s}$), and the transition from Ho↑ to Ho↓ was detected as a sudden jump in Δf, indicated by the black arrow in Fig. S9a. Afterward, the Ho↓ was measured from Δf by retracting the tip to its original tip sample distance ($18\,\text{s} \leq t \leq 21\,\text{s}$). Finally, the bias voltage was restored from $V = 200\,\mu\text{V}$ to its original value of $V = 120\,\text{mV}$ ($25\,\text{s} \leq t \leq 29\,\text{s}$).

In Fig. S9a, the minimum of Δf obtained on top of Ho↑ is smaller than that for Ho↓, indicating that the spin state of Ho can be read from Δf. Hence, Ho↑ can be switched to Ho↓ by approaching the tip. Moreover, in Fig. S9b, the averaged tunneling current after restoring the bias voltage ($t = 29$ s) is smaller than that before reducing the bias voltage ($t = 0$ s). These observations further support the spin switching from Ho↑ to Ho↓ by approaching the tip toward Ho_{top}.

10 Ho spin switching induced by tunneling current

Fig. S10(a–c) shows the time dependence of the tunneling current recorded over $\mathrm{Ho_{top}}$ at different tip–sample distances. The switching rates for various tip–sample distances are summarized in Fig. 4c. The histogram in Fig. S10d indicates a higher population of Ho$\uparrow$, revealing a preference for the state aligned with the external magnetic field.

11 Atom manipulation from $\mathrm{Ho_{top}}$ to $\mathrm{Ho_{bridge}}$ by tip approach

Here, we demonstrate that approaching the tip toward $\mathrm{Ho_{top}}$ induces atomic manipulation toward $\mathrm{Ho_{bridge}}$. Fig. S11a shows an STM topography image of three $\mathrm{Ho_{top}}$ atoms obtained in constant-current mode at $V = 200\,\mathrm{mV}$, confirming their initial adsorption sites. Among them, one $\mathrm{Ho_{top}}$ atom was selected as the target (gray arrow in Fig. S11a). For this target $\mathrm{Ho_{top}}$, the tip was positioned above the center of the atom, and its spin state was stabilized in the desired configuration (here, Ho$\downarrow$) by lowering the bias voltage from $V = 200\,\mathrm{mV}$ to $V = 200\,\mu\mathrm{V}$, well below the threshold for current-induced spin switching. Subsequently, the tip was approached vertically by decreasing the tip height until spin switching was observed (see Fig. S11b). The switching from Ho$\downarrow$ to Ho$\uparrow$ is evidenced by a sudden jump of about 0.2 Hz in Δf at $t = 19$ s (see Fig. S11c).

Next, after further approaching the tip toward $\mathrm{Ho_{top}}$ from the distance where spin switching occurred, the tip was held at a constant height (23 s $\leq t \leq$ 32 s). As a result, a sudden jump of approximately 0.8 Hz in Δf, corresponding to the atom manipulation from $\mathrm{Ho_{top}}$ to $\mathrm{Ho_{bridge}}$, was observed at $t = 28$ s (see Fig. S11c). Fig. S11d shows an STM image of the same scan area as Fig. S11a, recorded immediately after Fig. S11b in constant-current mode at $V = 200\,\mathrm{mV}$, revealing that the target $\mathrm{Ho_{top}}$ has transformed into a brighter spot corresponding to $\mathrm{Ho_{bridge}}$. These results demonstrates that approaching the tip to the center of $\mathrm{Ho_{top}}$ can induce a lateral displacement toward the bridge site, resulting in the formation of $\mathrm{Ho_{bridge}}$. Note that once the Ho atom relocates to the $\mathrm{Ho_{bridge}}$ position, it rarely returns to $\mathrm{Ho_{top}}$.

12 Imaging Ho adatom at small tip–sample distances

Fig. S12a and Fig. S12b show the Δf and conductance recorded on top of Ho$\uparrow$ and Ho$\downarrow$ as a function of tip–sample distance. To image Ho$\uparrow$ and Ho$\downarrow$ at small tip–sample distances, the tip height was set to $z = 0$ nm near the local minimum of Δf. Fig. S12c and Fig. S12d present Δf images of Ho$\uparrow$ and Ho$\downarrow$ acquired at $z = 0$ nm. In Fig. S12c, three distinct local minimum are observed on the Ho adatom (white dotted circle in Fig. S12c). As discussed in previous results, these three features indicate a three-atom tip [18]. Due to the asymmetric shape of the tip apex, atomic relaxation from $\mathrm{Ho_{top}}$ to $\mathrm{Ho_{bridge}}$ can be induced even when the tip is vertically approached toward the center of $\mathrm{Ho_{top}}$ (the center of $\mathrm{Ho_{top}}$ refers to the peak position of $\mathrm{Ho_{top}}$ in the STM image). Notably, spin switching from Ho$\downarrow$ to Ho$\uparrow$ was observed at $z = -0.11$ nm in Fig. S12a.

13 Force-based spin switching by different tips

To investigate the effect of tip shape on spin switching, we show the spin switching distance by approaching the Co tip at the center of Ho_{top} using different tip shapes. We prepared different tips using the method shown in Fig. S4 and examined the spin switching distance for each tip, corresponding to a fixed switching rate (here, 0.1 Hz). As shown in Fig. S13a, the spin switching distance varies significantly with tip shape. We further found that spin switching always occurs at distances shorter than the point-contact distance ($z < 0$). Therefore, we propose that atomic relaxation from Ho_{top} to Ho_{bridge} begins after the tip reaches point contact, and that spin switching occurs when the Ho atom moves laterally from the top site to some extent without fully reaching the bridge site. Fig. S13a confirms the reproducibility of the force-based spin switching.

14 Energy diagrams and spin switching under different magnetic fields

Fig. S14a presents the energy diagram for Ho_{top} calculated at $B = 1.0\,T$. The ground and metastable states are characterized by $\langle J_z \rangle = -8.0$ and $\langle J_z \rangle = +8.0$, respectively, with an energy splitting of 1.16 meV. These states are dominated by the $|m\rangle = \pm|8\rangle$ states with more than 99 % weight. No $\Delta m = \pm 1$ or $\Delta m = 0$ transitions are allowed between Ho↑ and Ho↓. In contrast, Fig. S14b shows the energy-level diagram for Ho_{bridge} at $B = 1.0\,T$. Here, the ground and metastable states exhibit $\langle J_z \rangle = -5.5$ and $\langle J_z \rangle = +5.5$. Notably, these states allow for a $\Delta m = 0$ transition with an energy difference of 1.2 meV. At $B = 8.0\,T$, Fig. S14c shows the diagram for Ho_{top}. The ground and metastable states are $\langle J_z \rangle = -8.0$ and $\langle J_z \rangle = +8.0$ with a larger energy splitting of 9.2 meV. These states are dominated by the $|m\rangle = \pm|8\rangle$ states with more than 99 % weight. No $\Delta m = \pm 1$ or $\Delta m = 0$ transitions are allowed between Ho↑ and Ho↓. Figure S14d displays the energy diagram for Ho_{bridge} at $B = 8.0\,T$. The ground and metastable states show $\langle J_z \rangle = -6.8$ and $\langle J_z \rangle = +6.6$, and they exhibit an allowed $\Delta m = 0$ transition with an energy difference of 7.8 meV. According to Fig.1b, Fig.S3b and Fig.S14(a-d), the spin reversal mechanism driven by the adsorption site change from top to bridge is qualitatively consistent at 1.0 T, 3.0 T, and 8.0 T. Therefore, we choose $B = 3.0\,T$ for the experimental measurements.

Figure S14(e–g) presents the observation of Ho spin switching at 8.0 T. As shown in the time-dependent Δf traces (Figs. S14e and f), clear spin switching is observed with a spin lifetime on the order of several seconds, similar to the behavior at 3.0 T. The tip–sample distance required to induce this switching is determined from the Δf curve in Fig. S14g, which reveals that the switching occurs when the tip–sample distance is reduced to a very close regime ($z \leq -143$ pm). Furthermore, the overall shape of the Δf curve in Fig. S14g is qualitatively similar to that shown in Fig. S8a. These findings are fully consistent with the results discussed in the main text, suggesting that the Ho spin switching at 8.0 T is governed by the same underlying mechanism as at 3.0 T—namely, a mechanism driven by a change in the adsorption site from a top site to a bridge site.

15 The Ho spin lifetime

Figure S15a demonstrates the effect of the tip–sample distance on spin stability. In the initial stage ($0\,\mathrm{s} \leq t \leq 49\,\mathrm{s}$), the Co tip is positioned at a relatively large tip–sample distance. In this regime, no spin switching occurs within 49 s. As the Co tip is brought closer to the sample ($t > 49$ s), the spin lifetime is reduced to several seconds, as evidenced by the random telegraph signal.

16 Proposed force-based spin switching mechanism

Here, we discuss two possible mechanisms of spin switching induced by force. The first possible mechanism involves spin–phonon coupling between Ho and the MgO substrate [8]. The Ho adatom on the MgO surface has a phonon density of states at approximately 4.7 meV [19]. Therefore, we propose that the relaxation from $\mathrm{Ho_{top}}$ to $\mathrm{Ho_{bridge}}$ allows spin switching, which can be induced by spin–phonon scattering. The second possible mechanism involves electron spin–spin scattering due to the proximity of the Co tip to the Ho atom [10]. The thermal energy of conduction electrons is estimated as $k_{\mathrm{B}}T \approx 0.388$ meV at 4.5 K. Thus, the relaxation from $\mathrm{Ho_{top}}$ to $\mathrm{Ho_{bridge}}$ allows spin switching, which can be induced by electron spin-spin scattering.

Additionally, we discuss the possibility that the oscillation of the magnetic tip could induce magnetic field sweeps across avoided level crossings, potentially leading to Landau–Zener tunneling (LZT) [20]. Hyperfine interactions create avoided level crossings, typically within a magnetic field range of approximately ± 200 mT [4]. Previous studies have demonstrated that, under zero external magnetic field, sweeping the magnetic field from the tip across these crossings can result in spin reversal [4]. However, in our experiment, we apply an external magnetic field of 3.0 T, which is far beyond the ± 200 mT range. Furthermore, the magnetization of the Co tip aligns with the external magnetic field. Consequently, the exchange field from the tip adds to the external field [21]. Thus, we conclude that the observed spin switching is not driven by LZT.

When the asymmetric Co tip is in contact with $\mathrm{Ho_{top}}$, we cannot exclude the possibility that the asymmetric Co tip may also reduce the symmetry of $\mathrm{Ho_{top}}$. However, because the Co tip is metallic and electrically neutral, whereas the oxygen and magnesium atoms in MgO are negatively and positively charged, the reduction of symmetry of $\mathrm{Ho_{top}}$ due to the crystal field is expected to be larger than that caused by the asymmetric tip.

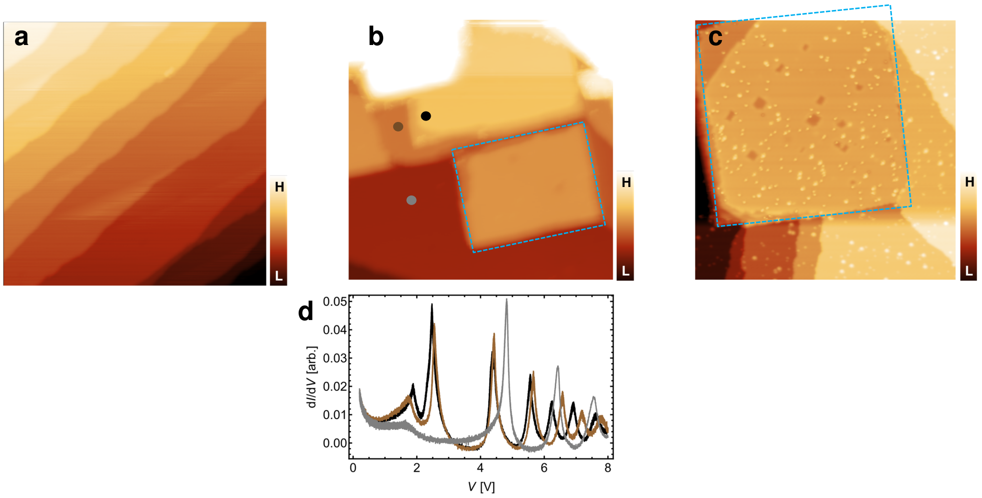

Fig. S1 STM images of Ag(001) and MgO/Ag(001). (a–c) STM topography images of Ag(001), MgO/Ag(001), and coadsorbed Ho and Co on MgO/Ag(001). The blue dotted rectangles indicate the MgO thin film. Imaging conditions: constant-current mode. (a) $I = 1.0\,\mathrm{nA}$, $V = 200\,\mathrm{mV}$, 50 nm × 50 nm; (b) $I = 1.0\,\mathrm{nA}$, $V = 200\,\mathrm{mV}$, scan size 100 nm × 100 nm; (c) $I = 200\,\mathrm{pA}$, $V = 400\,\mathrm{mV}$, scan size 80 nm × 80 nm. (d) Field emission spectra measured on Ag(001) (gray) and MgO/Ag(001) (brown and black) in constant-current mode ($I = 1\,\mathrm{nA}$). The spectroscopic locations are indicated by different colors in (b).

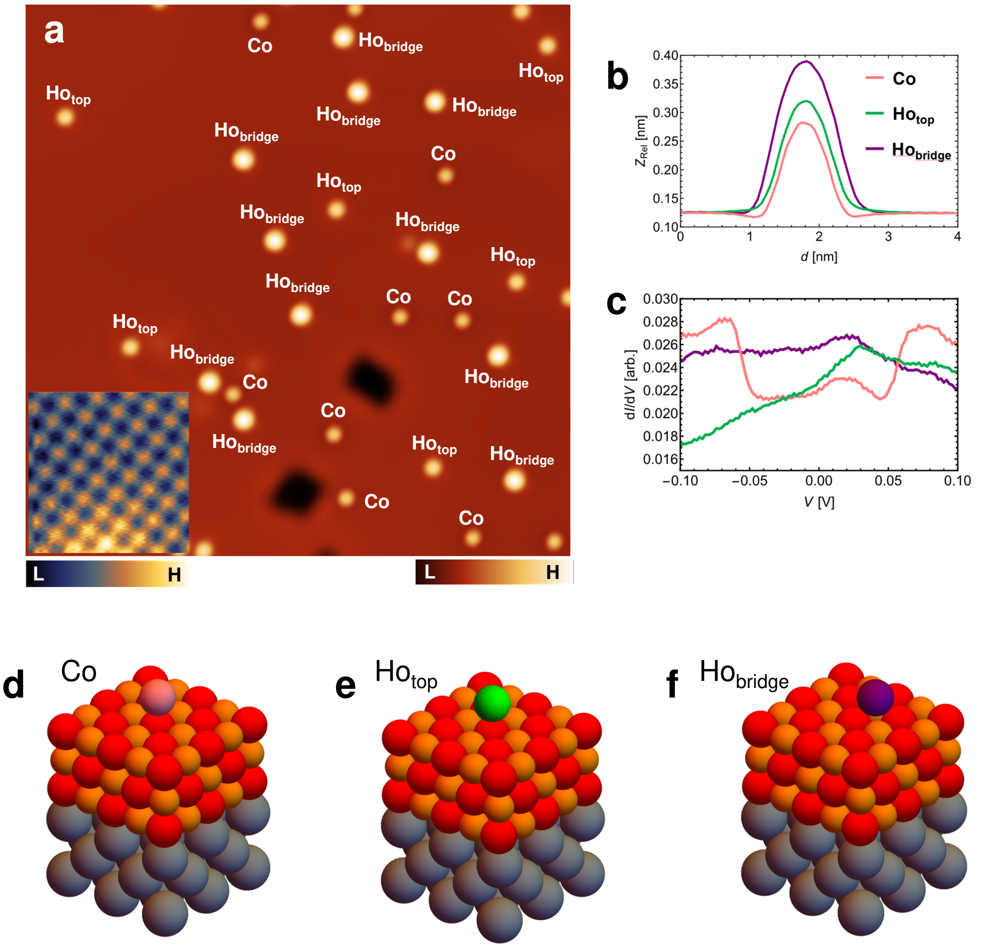

Fig. S2 Identification of Co, Ho$_{top}$ and Ho$_{bridge}$ (a) STM topography images of coadsorbed Ho and Co on MgO/Ag(001). The inset shows the atomically resolved Δf image of MgO thin film. Imaging condition for the STM topography image: constant current mode, $I = 1.0\,\text{nA}$, $V = 200\,\text{mV}$, $B = 3.0\,\text{T}$, scan size 30 nm × 30 nm. Imaging condition for the inset image: constant height mode, $V = 6.2\,\text{mV}$, $B = 3.0\,\text{T}$. (b) Line profile measured above Co, Ho$_{top}$ and Ho$_{bridge}$. (c) dI/dV spectrum measured on top of Co, Ho$_{top}$ and Ho$_{bridge}$. Same color code as (b). (d) Schematic of Co adatom located on MgO/Ag(001). (e,f) Schematic of Ho adatom located on top and bridge site. Pink ball: Co atom, green ball: Ho atom, purple ball: Ho atom, orange ball: Mg atom, red ball: O atom, gray ball: Ag atom in (d–f).

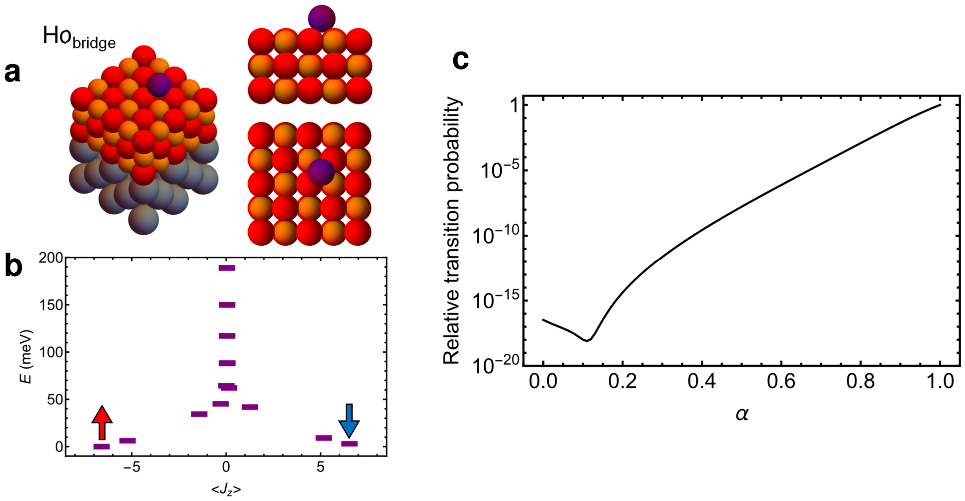

Fig. S3 Energy diagram of Ho$_{\mathrm{bridge}}$. (a) Three-dimensional, top, and side views of the adsorption configuration of a Ho atom at the bridge site in the low-symmetry C_{2v} position on MgO/Ag(100). Purple ball: Ho atom, orange ball: Mg atom, red ball: O atom, gray ball: Ag atom. (b) Calculated eigenvalues of bridge-site Ho in low-symmetry C_{2v} on MgO/Ag(100). Bridge-site Ho in low-symmetry C_{2v} configuration shows strongly superposed angular momentum states even at large magnetic fields of $B = 3.0\,\mathrm{T}$. The red and blue arrows in (b) indicate the ground state and metastable state (Ho↑ and Ho↓). In section 3 of the supporting information, the corresponding eigenvectors for Ho↑ and Ho↓ in (b) are denoted by $|\psi_0\rangle$ and $|\psi_1\rangle$, respectively. (c) A relative transition probability between the ground state and metastable state ($|\psi_0\rangle$ and $|\psi_1\rangle$) as a function of α.

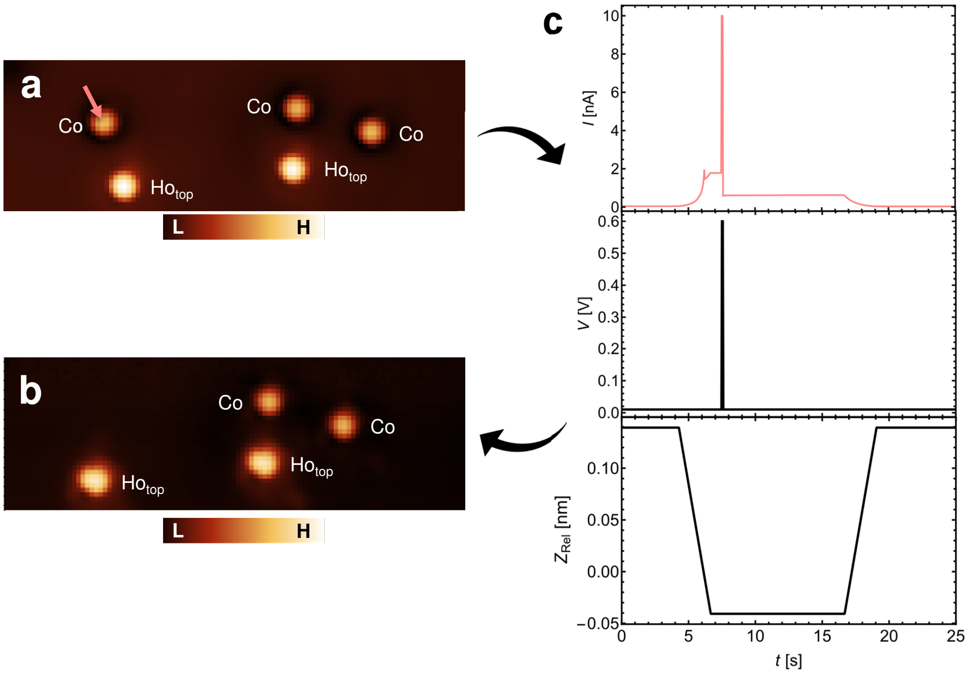

Fig. S4 Picking up a Co adatom from MgO/Ag(001). (a, b) Consecutive STM topography images of coadsorbed Ho_{top} and Co on MgO/Ag(001) before and after picking up Co from the surface. Imaging conditions: constant-current mode, $I = 1.0\,\mathrm{nA}$, $V = 200\,\mathrm{mV}$, $B = 3.0\,\mathrm{T}$, scan size 4.0 nm × 12 nm. (c) Time dependence of the tunneling current, bias voltage, and tip–sample distance during the pickup of a Co adatom from the surface.

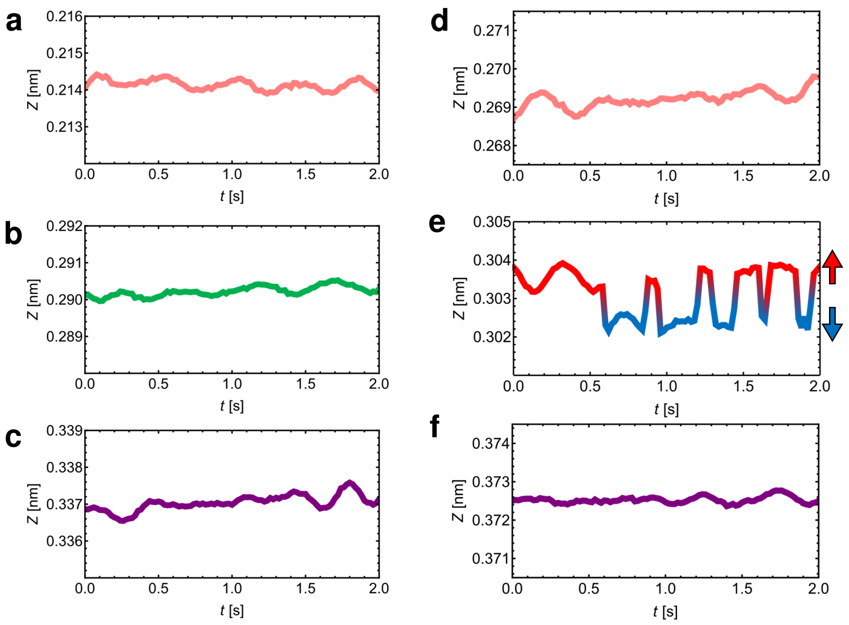

Fig. S5 Probing Co, $\mathrm{Ho_{top}}$, and $\mathrm{Ho_{bridge}}$ using non-magnetic tip and magnetic tip. (a–c) Time dependence of the tip–sample distance measured on top of Co, $\mathrm{Ho_{top}}$, and $\mathrm{Ho_{bridge}}$ using a non-magnetic tip. (d–f) Time dependence of the tip–sample distance measured on the same Co, $\mathrm{Ho_{top}}$, and $\mathrm{Ho_{bridge}}$ as in (a–c), immediately after picking up a Co adatom from the surface with the tip. The red and blue in (e) indicate Ho↑ and Ho↓. Measurement conditions: constant-current mode, $I = 1.0\,\mathrm{nA}$, $V = 200\,\mathrm{mV}$, $B = 3.0\,\mathrm{T}$.

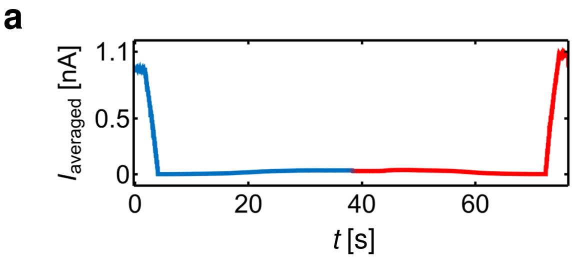

Fig. S6 Averaged tunneling current simultaneously obtained with Δf in Fig.2a. (a) Time evolution of averaged tunneling current, simultaneously recorded with Δf in Fig.2a. The blue and red correspond to the Ho$\downarrow$ and Ho$\uparrow$.

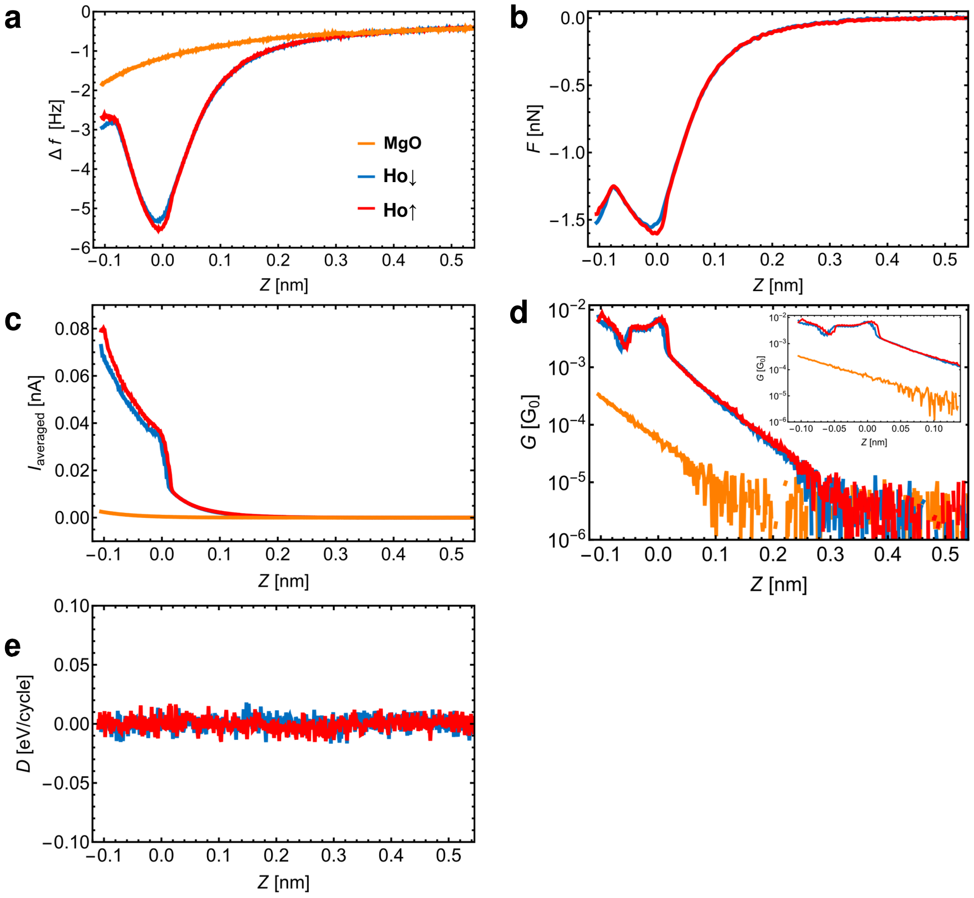

Fig. S7 Full data sets for Fig.3. (a) The distance dependence of frequency shift obtained on top of the Ho↑ ($\Delta f_{\mathrm{Ho}\uparrow}(z)$, red solid curve), Ho↓ ($\Delta f_{\mathrm{Ho}\downarrow}(z)$, blue solid curve) and MgO surface ($\Delta f_{\mathrm{MgO}}(z)$, orange solid curve). Measurement conditions: $V = 200\,\mu\mathrm{V}$ and $B = 3.0\,\mathrm{T}$. (b) Short-range force obtained on top of Ho↑ ($F_{\mathrm{Ho}\uparrow}(z)$, red) and Ho↓ ($F_{\mathrm{Ho}\downarrow}(z)$, blue). $F_{\mathrm{Ho}\uparrow}(z)$ and $F_{\mathrm{Ho}\downarrow}(z)$ were calculated from $\Delta f_{\mathrm{Ho}\uparrow\mathrm{SR}}(z) = \Delta f_{\mathrm{Ho}\uparrow}(z) - \Delta f_{\mathrm{MgO}}(z)$ and $\Delta f_{\mathrm{Ho}\downarrow\mathrm{SR}}(z) = \Delta f_{\mathrm{Ho}\downarrow}(z) - \Delta f_{\mathrm{MgO}}(z)$ [22]. (c) The distance dependence of the averaged tunneling current simultaneously recorded on top of Ho↑ (red solid curve), Ho↓ (blue solid curve) and MgO surface (orange solid curve) with Δf in (a). (d) The distance dependence of conductance at the lowest turnaround point of the tip oscillation cycle, obtained by deconvolving the time-averaged tunneling current in (c) [23]. Ho↑ (red solid curve), Ho↓ (blue solid curve) and MgO surface (orange solid curve) in (d). Inset shows the enlarged image of the range -0.11 nm $\leq z \leq 0.14$ nm. (e) Distance dependence of the dissipation simultaneously recorded with $\Delta f_{\mathrm{Ho}\downarrow}(z)$ and $\Delta f_{\mathrm{Ho}\uparrow}(z)$ in (a). Ho↑ (red solid curve) and Ho↓ (blue solid curve) in (e).

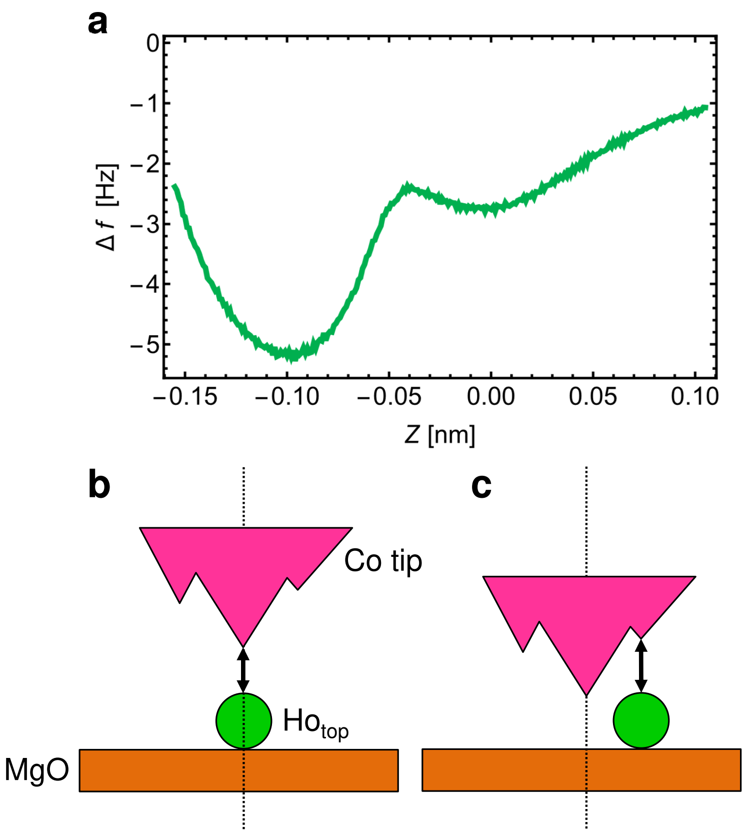

Fig. S8 $\Delta f(z)$ **measured on top of the Ho**$_{top}$. (a) The distance dependence of frequency shift obtained on top of the Ho adatom. (b,c) Schematic illustration of the lateral motion of the Ho adtom by approaching the tip. Large tip sample distance (b) and small tip sample distance (c). Measurement conditions: $V = 200\,\mu\mathrm{V}$ and $B = 3.0\,\mathrm{T}$.

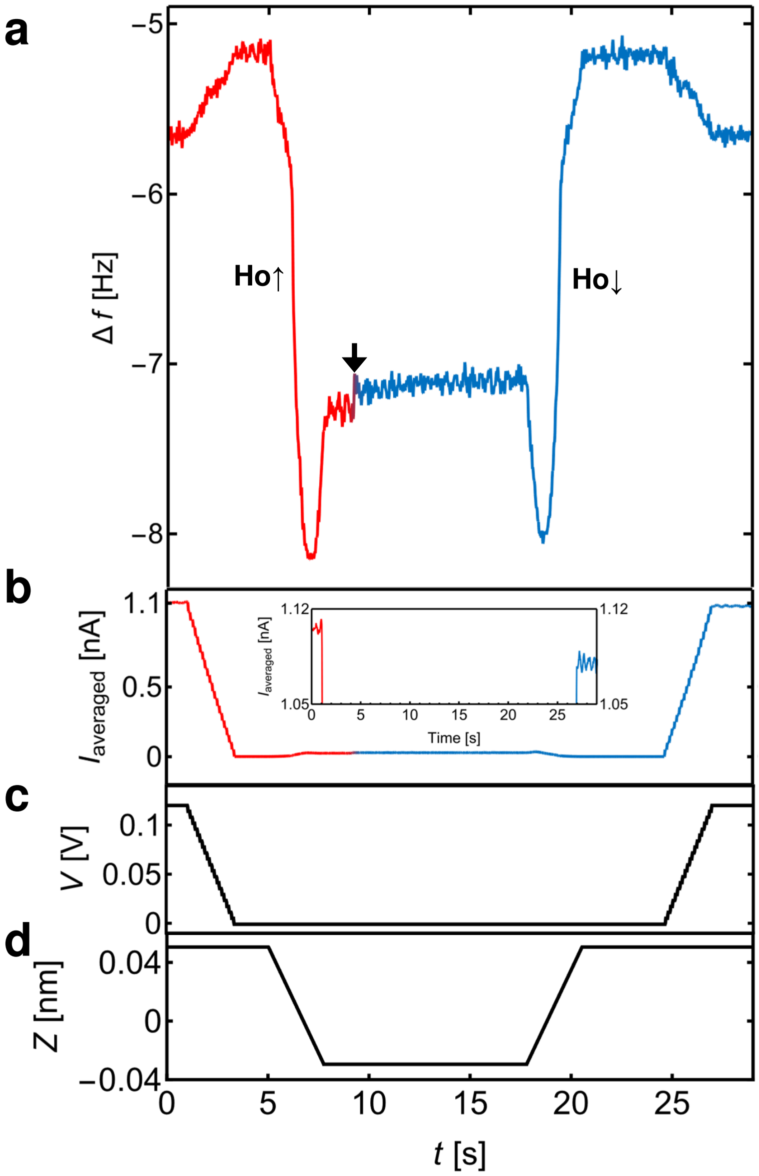

Fig. S9 Controlling Ho spin from Ho↑ to Ho↓ by approaching the tip. (a–d) $\Delta f(t)$ spectra (a) and averaged tunneling current (b) measured on top of $\mathrm{Ho_{top}}$ while varying the bias voltage (c) and the tip sample distances (d). The red and blue in (a,b) indicate the Ho↑ and Ho↓. At 8 s $\leq t \leq$ 18 s, the transition from the Ho↑ to Ho↓ state can be detected by a sudden jump in $\Delta f(t)$, marked by the black arrow. The inset in (b) shows the enlarged image of the averaged tunneling current at $t = 0$ s and $t = 29$ s. Measurement conditions: $B = 3.0\,\mathrm{T}$.

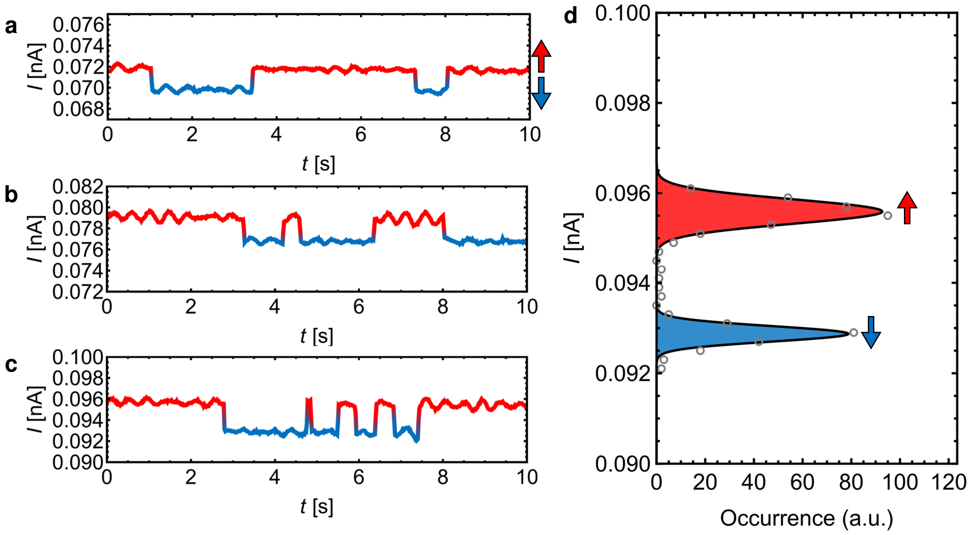

Fig. S10 Spin switching induced by tunneling current. (a–c) Time dependence of the tunneling current measured on top of $\mathrm{Ho_{top}}$ while varying the tip–sample distance. The red and blue indicate the Ho↑ and Ho↓. Measurement conditions: constant-height mode, $V = 150\,\mathrm{mV}$, $B = 3.0\,\mathrm{T}$, (a) $z = 292\,\mathrm{pm}$; (b) $z = 287\,\mathrm{pm}$; (c) $z = 277\,\mathrm{pm}$. (d) Current histogram in (c). The gray circles represent the experimental data, and the black solid curve shows the Gaussian fit to the experimental data.

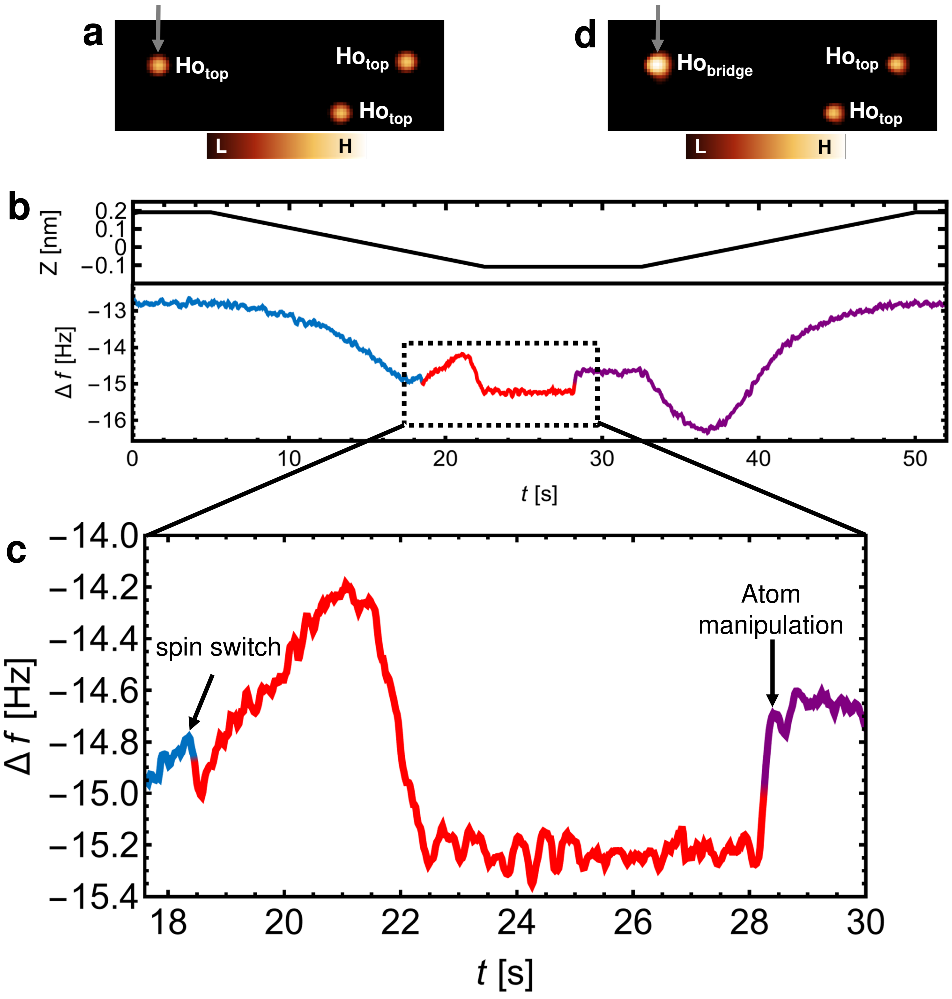

Fig. S11 Atom manipulation induced by approaching the tip. (a) STM topography image of Ho_{top} on MgO/Ag(001). The gray arrow indicates the target Ho_{top}. Imaging conditions: constant-current mode, $I = 1.0\,nA$, $V = 200\,mV$, $B = 3.0\,T$, scan size 4.2 nm × 14 nm. (b) Time dependence of Δf as a function of tip–sample distance. The blue, red and purple indicate the Ho↓, Ho↑ and Ho_{bridge}. Measurement conditions: $V = 200\,\mu V$ and $B = 3.0\,T$. (c) Enlarged view of (b) for 17 s $\leq t \leq$ 30 s, showing a sudden change in Δf corresponding to spin switching and atom manipulation from Ho_{top} to Ho_{bridge}. (d) STM topography image of the same area as in (a), acquired immediately after (b). Imaging conditions: constant-current mode, $I = 1.0\,nA$, $V = 200\,mV$, $B = 3.0\,T$, scan size 4.2 nm × 14 nm.

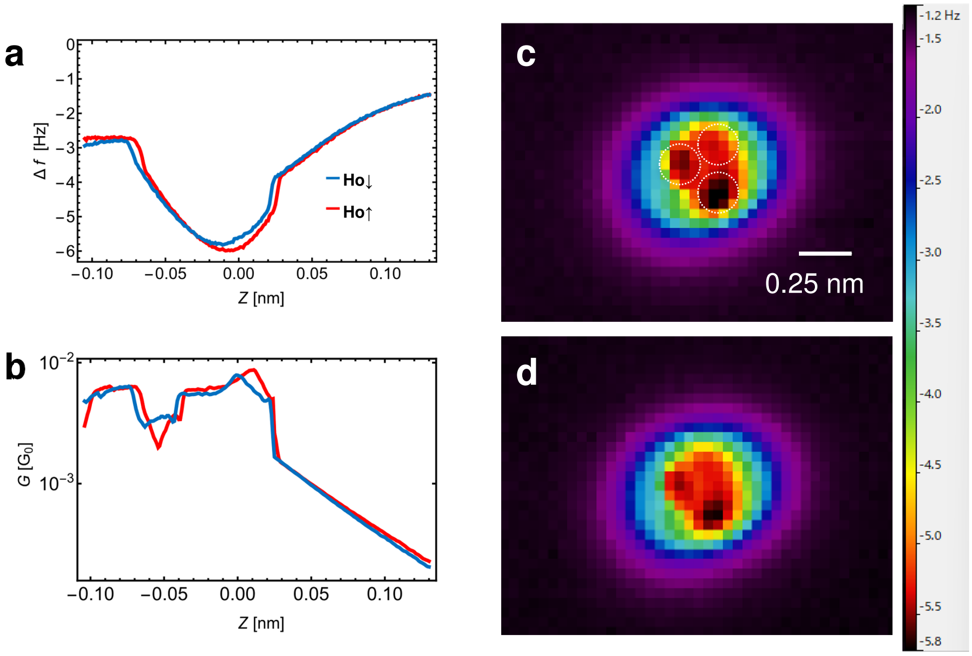

Fig. S12 Imaging Ho adatom at small tip–sample distances. (a,b) The distance dependence of frequency shift and conductance obtained on top of the Ho↑ ($\Delta f_{\mathrm{Ho}\uparrow}(z)$, red solid curve) and Ho↓ ($\Delta f_{\mathrm{Ho}\downarrow}(z)$, blue solid curve). Measurement conditions: $V = 200\,\mu\mathrm{V}$ and $B = 3.0\,\mathrm{T}$. Conductance is at the lowest turnaround point of the tip oscillation cycle obtained from the average tunneling current. (c,d) Δf images of Ho↑ and Ho↓. Imaging parameter: constant height mode. $z = 0\,\mathrm{nm}$, $V = 200\,\mu\mathrm{V}$, $B = 3.0\,\mathrm{T}$, 1.5 nm × 1.7 nm. White dotted circles in (c) indicate the three local minima of Δf in the Ho adatom.

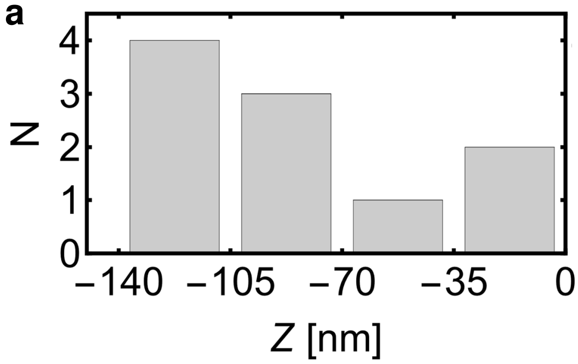

Fig. S13 Force-based spin switching induced by different tip. (a) Histogram of tip–sample distance of spin switching using different tip shapes.

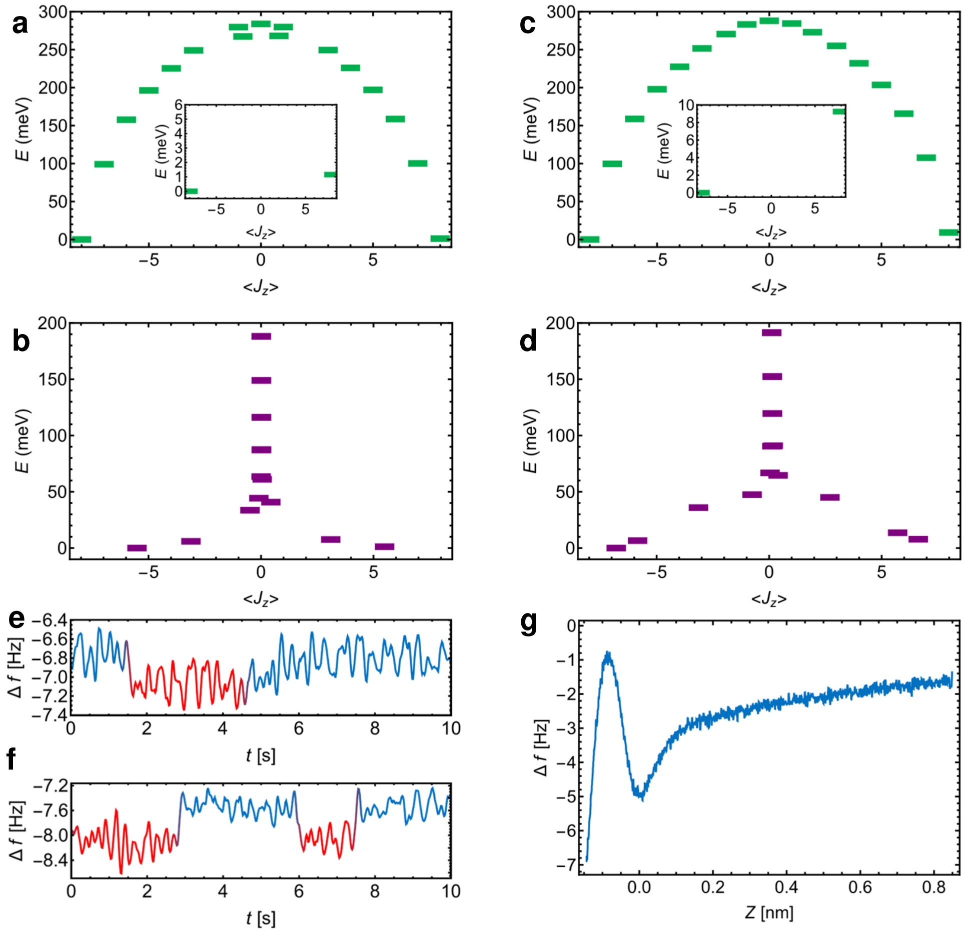

Fig. S14 Calculated eigenvalues of Ho on MgO/Ag(100). (a) Top site at 1.0 T. (b) Bridge site at 1.0 T. (c) Top site at 8.0 T. (d) Bridge site at 8.0 T. The insets in (a) and (c) show a magnified view of the low-energy region. (e,f) Time dependence of Δf measured on top of $\mathrm{Ho_{top}}$ while varying the tip–sample distance. The red and blue indicate the Ho↑ and Ho↓. Measurement conditions: constant-height mode, $V = 4\,\mathrm{mV}$, $B = 8.0\,\mathrm{T}$, (e) $z = -143\,\mathrm{pm}$ and (f) $z = -149\,\mathrm{pm}$. (g) The distance dependence of Δf obtained on top of the Ho↓. Measurement conditions: $V = 4\,\mathrm{mV}$, $B = 8.0\,\mathrm{T}$. Note that the data in (e–g) were acquired using a home-made length-extension resonator (LER) equipped with an Au tip. A magnetic tip was prepared by picking up Co atoms. The oscillation amplitude was set to 30 pm.

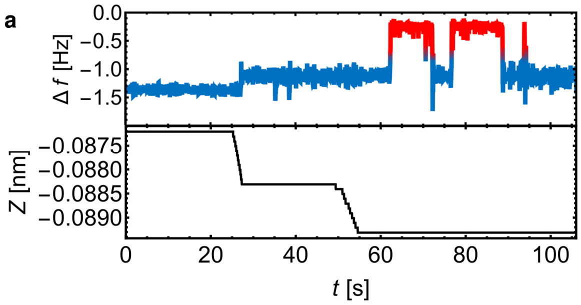

Fig. S15 The Ho spin lifetime. (a) Time dependence of Δf as a function of tip–sample distance. The blue and red indicate the Ho↓ and Ho↑. Measurement conditions: $V = 200\,\mu\mathrm{V}$ and $B = 3.0\,\mathrm{T}$.

References

[1] Singha, A., Donati, F., Natterer, F.D., Wäckerlin, C., Stavrić, S., Popović, Z.S., Šljivančanin, Ž., Patthey, F., Brune, H.: Spin excitations in a $4f$ - $3d$ heterodimer on MgO. Physical review letters **121**(25), 257202 (2018)

[2] Rau, I.G., Baumann, S., Rusponi, S., Donati, F., Stepanow, S., Gragnaniello, L., Dreiser, J., Piamonteze, C., Nolting, F., Gangopadhyay, S., *et al.*: Reaching the magnetic anisotropy limit of a $3d$ metal atom. Science **344**(6187), 988–992 (2014)

[3] Natterer, F.D., Donati, F., Patthey, F., Brune, H.: Thermal and magnetic-field stability of holmium single-atom magnets. Physical review letters **121**(2), 027201 (2018)

[4] Forrester, P.R., Patthey, F., Fernandes, E., Sblendorio, D.P., Brune, H., Natterer, F.D.: Quantum state manipulation of single atom magnets using the hyperfine interaction. Physical Review B **100**(18), 180405 (2019)

[5] Chen, Y., Liu, J., Ju, M., Qiu, R., Yuan, H.: Magnetic stability of Ce and Nd single atom magnets on insulating MgO/Ag (100). Physical Review B **107**(21), 214444 (2023)

[6] Freeman, A.J., Watson, R.: Theoretical investigation of some magnetic and spectroscopic properties of rare-earth ions. Physical Review **127**(6), 2058 (1962)

[7] Stevens, K.: Matrix elements and operator equivalents connected with the magnetic properties of rare earth ions. Proceedings of the Physical Society. Section A **65**(3), 209 (1952)

[8] Bilgeri, T.: Quantum dynamics in individual surface spins. PhD thesis, EPFL (2021)

[9] Natterer, F.D., Yang, K., Paul, W., Willke, P., Choi, T., Greber, T., Heinrich, A.J., Lutz, C.P.: Reading and writing single-atom magnets. Nature **543**(7644), 226–228 (2017)

[10] Ternes, M.: Spin excitations and correlations in scanning tunneling spectroscopy. New Journal of Physics **17**(6), 063016 (2015)

[11] Otte, A.F.: Magnetism of a single atom. PhD thesis, Leiden Universiteit (2008)

[12] Baumann, S.: Investigation of the unusual magnetic properties of Fe and Co on MgO with high spatial, energy and temporal resolution. PhD thesis, Universit¨at Basel (2015)

[13] Ternes, M., González, C., Lutz, C.P., Hapala, P., Giessibl, F.J., Jelínek, P., Heinrich, A.J.: Interplay of conductance, force, and structural change in metallic point contacts. Physical review letters **106**(1), 016802 (2011)

[14] Kröger, J., Néel, N., Limot, L.: Contact to single atoms and molecules with the tip of a scanning tunnelling microscope. Journal of Physics: Condensed Matter **20**(22), 223001 (2008)

[15] Paul, W., Yang, K., Baumann, S., Romming, N., Choi, T., Lutz, C.P., Heinrich, A.J.: Control of the millisecond spin lifetime of an electrically probed atom. Nature Physics **13**(4), 403–407 (2017)

[16] Schirmeisen, A., Weiner, D., Fuchs, H.: Single-atom contact mechanics: From atomic scale energy barrier to mechanical relaxation hysteresis. Physical review letters **97**(13), 136101 (2006)

[17] Bamidele, J., Li, Y., Jarvis, S., Naitoh, Y., Sugawara, Y., Kantorovich, L.: Complex design of dissipation signals in non-contact atomic force microscopy. Physical Chemistry Chemical Physics **14**(47), 16250–16257 (2012)

[18] Gretz, O., Weymouth, A.J., Giessibl, F.J.: Identifying the atomic configuration of the tip apex using stm and frequency-modulation afm with CO on Pt (111). Physical Review Research **2**(3), 033094 (2020)

[19] Donati, F., Rusponi, S., Stepanow, S., Persichetti, L., Singha, A., Juraschek, D.M., Wäckerlin, C., Baltic, R., Pivetta, M., Diller, K., *et al.*: Unconventional spin relaxation involving localized vibrational modes in Ho single-atom magnets. Physical review letters **124**(7), 077204 (2020)

[20] Zener, C.: Non-adiabatic crossing of energy levels. Proceedings of the Royal Society of London. Series A, Containing Papers of a Mathematical and Physical Character **137**(833), 696–702 (1932)

[21] Yang, K., Paul, W., Natterer, F.D., Lado, J.L., Bae, Y., Willke, P., Choi, T., Ferrón, A., Fernández-Rossier, J., Heinrich, A.J., *et al.*: Tuning the exchange bias on a single atom from 1 mT to 10 T. Physical review letters **122**(22), 227203 (2019)

[22] Sader, J.E., Jarvis, S.P.: Accurate formulas for interaction force and energy in frequency modulation force spectroscopy. Applied Physics Letters **84**(10), 1801–1803 (2004)

[23] Sader, J.E., Sugimoto, Y.: Accurate formula for conversion of tunneling current in dynamic atomic force spectroscopy. Applied Physics Letters **97**(4), 043502 (2010)
