# Supplementary material for: Force-based reading and writing of individual single-atom magnets
Source: Nat Commun. 2026 Jul 9;17:5927. doi: 10.1038/s41467-026-74922-z (PMC13350084; doi:10.1038/s41467-026-74922-z)
Supplement: Supplementary file 2 — Peer Review File [file 41467_2026_74922_MOESM2_ESM.pdf]

nature portfolio

# Peer Review File

# Force-Based Reading and Writing of Individual Single-Atom Magnets

Corresponding Author: Dr Yoshiaki Sugimoto

**Parts of this Peer Review File have been redacted as indicated to remove third-party material.**

**This file contains all reviewer reports in order by version, followed by all author rebuttals in order by version.**

Version 0:

Reviewer comments:

Reviewer #1

(Remarks to the Author)
The manuscript of Yuuki Adachi et al. reports on the use of magnetic exchange force microscopy to read and write the spin orientation of a single holmium adatom on MgO thin films. Using an atomic force to control and probe the quantum states of an atomic-scale quantum matter can be a very promising alternative to avoid the inherent drawback of using a scanning tunneling microscope that uses tunneling current and resultant inevitable energy dissipation and decoherence, as the authors mentioned in the page 4, lines 160-163 "Spin detection and manipulation of 4f-electron systems by a non-dissipative force, unlike electric currents, is expected to lead to the realization of long spin coherence times, which are critical for quantum information processing." The experimental results are of a very high quality, and the manuscript is well written, though some clarifications/improvements will be needed. Overall, the implementation of force-based control/probe of the quantum spin states of a single atom will be highly relevant for the design of future artificial spin systems with atomic precision, further, the topic is highly relevant for an atomic scale engineering of spin qubits for the realization of various quantum platforms on material surfaces.

This report is very novel and timely, thus I recommend the manuscript for publication in Nature Communications after a revision considering the following points:

MAJOR:

- The reversible switching clearly supported that z_tip dependent exchange force is not the major driving force of the switching as the authors mentioned in the page 3, lines 117-118. I personally think that the result here can be called "spin-switching of a single atom, driven by strain-induced state mixing using an atomic probe". Whatever the naming, the authors should state this aspect of the work in a solid manner throughout the paper. In the same sense, the proposition in the page 4, lines 139-144 doesn't sound convincing enough. I believe the discussion on the driving force of the switching should be improved.

- All the crucial parts of the experiments, e.g., exchange coupling, switching have been performed in a regime of direct contact between the tip and Ho atom (z < 0), where frequency shift (Delta_f) is the signal read out. But Delta_f relevant to the interaction with the local interaction at a specific tip position is conventionally described in a model of "non-contact" operation regime of AFM. Here, the FM mode operation at a "contact" regime and the relevance between Delta_f and tip-sample interaction is not intuitively understandable. This point should be more kindly described in the page 2, lines from 60.

- A questionable point to me is that the authors are sure to exclude the influence of the tunneling electrons on the switching in the z regime (< 0) and bias voltage of 0.2 mV (page 2, lines from 60). In this point contact regime, quantum conductance should be considered (see Ref. [W. Paul et al. Nat. Phys. 13, 403–407 (2017)]). An in-depth discussion on this point can make the force-induced switching more convincing.

- The authors briefly mentioned intra- and inter-atomic couplings at a contact regime as the origin of the crossover between FM and AFM tip-Ho interaction (page 3, lines 99-105). As the experimental z-trace data shown in Fig.3 clearly revealed the crossover, the authors doesn't have to rely on the previous works but can give a detailed discussion on the specific case in this work (Co tip and Ho_top atom), which would strengthen the impact of the current work.

MINOR:

- Superparamagnetic nature of a magnetic tip apex has been thoroughly studied in the works [Appl. Phys. Lett. 103, 032407 (2013)] and [Nano Convergence 4, 8 (2017)], which can be referred in the page 2, line 59.

- A clear example of "controlling the spin by manipulating the adsorption-site symmetry" (page 4, lines 158-159) has been recently published: [Nat. Commun. (2026), https://www.nature.com/articles/s41467-026-68314-6].

- Fig.1b: A finite external magnetic field lifts the degeneracy of the two zero-field ground states (indicated by the red and blue arrows), and the authors try to show it in the figure, but it is not that visible in the energy scale of ~300 meV, including the whole eigenstates of the system. How about using an inset just for the two states of the interest using an energy scale, comparable to the Zeeman energy splitting of the states? The central part of Fig.1b looks pretty empty.

Reviewer #2

(Remarks to the Author)
Adachi et al. report force-based reading and writing of magnetic states of Ho adatoms on MgO using magnetic exchange force microscopy. The authors propose that mechanical manipulation offers a low-dissipation and highly localized alternative to conventional electrical reading and writing of single-atom magnets. Although the experiments are technically demanding and carefully carried out, it remains unclear whether the work constitutes a significant advance to justify publication in Nature Communications.

In particular, the manuscript does not convincingly demonstrate that mechanical readout and writing provide a clear conceptual or practical advantage over electrical reading and writing schemes (as in Ref. 5). The force-based readout requires probing times of several seconds to tens of seconds due to the slow tip approach, whereas electrical readout based on tunnel current in STM-based schemes can be performed on much shorter timescales. This slow operation may limit the practical relevance of the mechanical approach for memory applications. If the tip could approach very fast to reduce the readout time, it may induce spin transitions which disturbs the Ho state.

The reading method also appears intrinsically invasive. The reading relies on strong tip-atom interactions, including strong magnetic fields from the Co-functionalized tip and the scattering electrons from the tip apex. As a result, the measured lifetime using forces (several seconds) is comparable to the current-based STM measurement.

While the authors argue that electrical currents lead to Joule heating, this effect on reading and writing atoms is typically negligible under standard STM operating conditions (from several hundred millikelvin to a few kelvin).

Several additional points would benefit from clarification:
1. The experiments were performed at very small tip-atom distances, close to or beyond the point-contact regime. Under these conditions, changes in the tip apex configuration are likely during readout. If so, it could make it difficult to reliably distinguish the two Ho spin states, as the frequency shift depends sensitively on the detailed tip configuration.
2. The claim that force-induced switching is “more spatially localized” because the switching rate decays more rapidly with distance (Fig. 4c) is not fully convincing. The measured decay mainly reflects the vertical dependence of the interaction. However, addressability in dense atomic arrays is determined by the lateral extent of the perturbation. A short vertical decay does not necessarily imply narrow lateral control, particularly for mechanically mediated interactions and magnetic stray fields.
3. The transition from ferromagnetic to antiferromagnetic coupling is mentioned but not clearly explained. Adding one or two brief sentences to outline the physical origin of this transition would improve clarity and help readers follow the discussion without having to consult the cited literature in detail.

Reviewer #3

(Remarks to the Author)
The work “Force-Based Reading and Writing of Individual Single-Atom Magnets” by Adachi and coworkers reports on the timely investigation of the magnetic switching of single atom magnets using an atomic force microscope. The results are interesting because holmium single atom magnets on MgO have been shown to exhibit unusual magnetic bistability and their reading and writing have been largely based on current pulses using a scanning tunneling microscope and magnetic field sweeps.
In their work, Adachi and coworkers claim that they can read and write these single atom magnets by force interaction with the tip of an atomic force microscope. Their rationale is that the Ho atoms in the MgO top site (residing above an O atom) have magnetic bistability but Ho adatoms on MgO bridge site exhibit mixed ground states. They claim that by bringing the tip close enough to the Ho atom, it slightly shifts position from top to bridge, essentially moving from the bistable to the mixed state and back which may result in a spin flip/state reversal.

While we follow and tend to agree with the reading part using non-contact force microscopy, showing nice frequency shift contrast between the Ho-up and Ho-down states, the current evidence does not yet substantiate force-based writing. Looking at previous reports on magnetic switching of Ho single atom magnets, it appears essential to rule out the magnetic field sweep via the hyperfine coupled levels as the cause for state reversals. Presently, Adachi and colleagues use

magnetic tips and oscillations amplitudes of 65 pm that could randomize the states via Landau Zener tunneling, as the motion of a magnetic field is akin to performing magnetic field sweeps, as was previously shown. The claimed force-based switching would accordingly require using truly nonmagnetic tips, performing the partial manipulation of the Ho atom from top towards the bridge site and then checking the Ho state using a magnetic read out, either via switching analysis, frequency shift, or ESR. State reversals may indeed occur in the authors' experiment; however, the present data do not isolate force-based switching from magnetic-field–induced transitions.

In the light of these reservations and because force-based switching is prominently encoded in the title, we advise against publication in the present form until this major item is properly addressed. To substantiate force-based writing, the authors must demonstrate reversible state control using a nonmagnetic tip, exclude magnetic-field–induced transitions via quantitative modeling of the tip field, and provide external-field and hyperfine-resolved switching statistics. We also mention here that substantial experimental evidence is relayed to the supplement. We believe that all major claims need to be sufficiently corroborated within the main part of the article already.

We also have smaller reservations listed below that would warrant revisions.

Items in no particular order:
-no justification given for the choice of 3T magnetic biasing field
-Ho adatoms would not be stable at zero field without hyperfine interaction, which is not addressed by Adachi
-oscillation amplitude only cited in figure captions and not in experimental section
-electronic switching threshold around 73meV with transverse and of order and below 120meV without transverse magnetic field, so using 120mV to monitor Ho state could already lead to current induced state reversal
-Adachi and coworkers mention "exceeding threshold" for instance on p1L68 but do not clarify what parameters they are referring to.
-Fig 4. Redundant panels (a) and (b)
-no mention of exchange and dipolar magnetic fields created by the cobalt functionalized tip
-missing switching histogram that should reveal state preference for Ho state aligned with external magnetic field
-no discussion of energetics Ho state aligned with magnetic field has a meV preference to the opposite state
-Stevens parameters from SI differ from cited parameters for J=8
-discussion of Landau Zener tunneling missing
-no dependence on external magnetic field shown
-claims of higher spatial switching resolution not clear
-role of hyperfine interaction

Version 1:

Reviewer comments:

Reviewer #1

(Remarks to the Author)
The authors clarified point-by-point all the comments from me and revised the manuscript in appropriate ways. The revision is now in a good shape for publication in Nature Communications. I recommend publication of the manuscript as it is.

Reviewer #2

(Remarks to the Author)
The authors have addressed several of the previous comments, and the manuscript has improved. However, a few points would still benefit from further clarification.

1. The authors state that "we believe that the spin lifetime during readout remains long." Does Fig. 4a correspond to the reading or writing process? If it is a reading, the lifetime is only several seconds, which does not seem particularly long compared with the current-based readout scheme. For clarity, it would be useful to display the frequency-shift signals for both the readout and writing processes in the same figure. In addition, what is the longest spin lifetime that can be achieved using the force-based readout method?

2. The statement that "In this near-contact regime, electron scattering from the tip apex is suppressed." may be incorrect. The scattering should increase exponentially with decreasing tip-atom distance [Nat. Phys. 13, 403 (2017)]]. Therefore, electron scattering in the near-contact regime would be expected to be significantly stronger than in the current-based readout scheme used in Ref. 5. In this sense, the proposed readout method may also be intrinsically invasive. It would be helpful if the authors could further clarify this point.

Reviewer #3

(Remarks to the Author)
The authors have attempted to reply to the comments raised by the reviewers. While some have been satisfactory, we remain not convinced by the response to the major items and request further clarification.

The main issue is the unsatisfactory discussion of Landau Zener tunneling as a potential switching mechanism in their AFM investigation of the Ho single atom magnets. The statement that 3T is far from the hyperfine levels is misleading. While it may be true that 3T is large enough stabilize the Ho adatom; this holds true only in absence of the tip or when the tip-sample separation is large. However, the supposed force based switching requires a close proximity of the tip-apex with the Ho adatom and the Ho adatom therefore experiences a different effective field than it that simple picture. The authors must prove and rule out that the essential point contact between Co and Ho does not cause a different scenario. This close separation of a magnetic tip with a magnetic adatom is the regime where exchange forces dominate and have been shown to easily exceed 3T, for instance Yang, Phys. Rev. Lett. 122, 227203 (2019). We have already suggested a potential experimental verification that the authors dismissed. Here is another one: The authors could repeat the experiment at zero field, where Ho adatoms are stable and attempt to reverse the state at close proximity, where the exchange fields created by Co tip may now permanently move the Ho state towards a stable configuration for the writing period and the resulting state reversal would then be more closely related to force rather than to LZT. Alternatively, repeat the experiment at even more extreme fields and investigate whether the switching dynamics is field dependent.

One of the additional items mentioned by the authors is that the suggested force based magnetic switching could be applied to nonconducting surfaces. While it may be true, this is not necessarily a unique feature of a supposed force based reversal as the field-sweep technique introduced by Forrester Phys. Rev. B 100, 180405(R) (2019) already covers non conducting substrates. The latter field-sweep is incidentally noninvasive and can be made selective with appropriately sharp field gradients.

Unless these issues are properly addressed, we refrain from providing support for publication.

Version 2:

Reviewer comments:

Reviewer #2

(Remarks to the Author)
The authors have addressed my comments. I recommend publication.

I have read the review and reply, and here is my comment.

As pointed out by Reviewer #3, the oscillating magnetic field generated by the tip could in principle induce Ho switching under an external magnetic field. The authors rule out this possibility by measuring the Ho switching at a higher external field (8 T), where they observe behavior similar to that at lower magnetic fields.

However, at such a short tip-atom distance, the tip field arising from exchange interaction is expected to dominate over the dipolar stray field. Therefore, the statement in the SI, “Furthermore, the magnetization of the Co tip aligns with the external magnetic field. Consequently, the stray field from the tip adds to the external field [27],” may not be fully accurate, as it appears to refer mainly to the dipolar contribution. I suggest the authors also discuss the role of oscillating exchange field in the Ho switching process.

**Open Access** This Peer Review File is licensed under a Creative Commons Attribution 4.0 International License, which permits use, sharing, adaptation, distribution and reproduction in any medium or format, as long as you give appropriate credit to the original author(s) and the source, provide a link to the Creative Commons license, and indicate if changes were made.
In cases where reviewers are anonymous, credit should be given to 'Anonymous Referee' and the source.
The images or other third party material in this Peer Review File are included in the article’s Creative Commons license, unless indicated otherwise in a credit line to the material. If material is not included in the article’s Creative Commons license and your intended use is not permitted by statutory regulation or exceeds the permitted use, you will need to obtain permission directly from the copyright holder.
To view a copy of this license, visit https://creativecommons.org/licenses/by/4.0/

We would like to thank the referees for their careful reading of the manuscript and for providing many constructive comments. In response, we have revised several descriptions and addressed all the comments raised. The corresponding changes are highlighted in red in the revised manuscript, as detailed below. We hope that the revised version is now clearer for readers and suitable for publication in *Nature Communications*.

On behalf of all authors,

Yoshiaki Sugimoto

---

# Reviewer 1

**Reviewer Comment 1.1** — The manuscript of Yuuki Adachi et al. reports on the use of magnetic exchange force microscopy to read and write the spin orientation of a single holmium adatom on MgO thin films. Using an atomic force to control and probe the quantum states of an atomic-scale quantum matter can be a very promising alternative to avoid the inherent drawback of using a scanning tunneling microscope that uses tunneling current and resultant inevitable energy dissipation and decoherence, as the authors mentioned in the page 4, lines 160-163 "Spin detection and manipulation of $4f$-electron systems by a non-dissipative force, unlike electric currents, is expected to lead to the realization of long spin coherence times, which are critical for quantum information processing." The experimental results are of a very high quality, and the manuscript is well written, though some clarifications/improvements will be needed. Overall, the implementation of force-based control/probe of the quantum spin states of a single atom will be highly relevant for the design of future artificial spin systems with atomic precision, further, the topic is highly relevant for an atomic scale engineering of spin qubits for the realization of various quantum platforms on material surfaces.

This report is very novel and timely, thus I recommend the manuscript for publication in Nature Communications after a revision considering the following points:

**Reply**: We sincerely thank the reviewer for their encouraging comments and for recognizing the high quality of our experimental results regarding magnetic exchange force microscopy on single holmium adatoms. We are particularly pleased that the reviewer appreciates the significance of our non-dissipative force-based approach which avoids electric currents, and its relevance to future atomic-scale quantum engineering.

**Reviewer Comment 1.2** — MAJOR: The reversible switching clearly supported that $z_{\text{tip}}$ dependent exchange force is not the major driving force of the switching as the authors mentioned in the page 3, lines 117-118. I personally think that the result here can be called

"spin-switching of a single atom, driven by strain-induced state mixing using an atomic probe". Whatever the naming, the authors should state this aspect of the work in a solid manner throughout the paper. In the same sense, the proposition in the page 4, lines 139-144 doesn't sound convincing enough. I believe the discussion on the driving force of the switching should be improved.

**Reply**: We appreciate the reviewer's insightful comment regarding the driving force of the switching mechanism. Following this suggestion, we have added the proposition as follows

Page 4 of the main text *"We therefore propose that spin-switching of a single atom is driven by strain-induced state mixing using an atomic probe."*

**Reviewer Comment 1.3** — All the crucial parts of the experiments, e.g., exchange coupling, switching have been performed in a regime of direct contact between the tip and Ho atom ($z < 0$), where frequency shift ($\Delta f$) is the signal read out. But $\Delta f$ relevant to the interaction with the local interaction at a specific tip position is conventionally described in a model of "non-contact" operation regime of AFM. Here, the FM mode operation at a "contact" regime and the relevance between $\Delta f$ and tip-sample interaction is not intuitively understandable. This point should be more kindly described in the page 2, lines from 60.

**Reply**: We appreciate the reviewer for raising this important point regarding the interpretation of the frequency shift ($\Delta f$) in the contact regime. In frequency modulation atomic force microscopy (FM-AFM), $\Delta f$ reflects the gradient of the force between the tip and the sample. This relationship is conventionally described as:

$$\Delta f \approx -\frac{f_0}{2k}\frac{\partial F}{\partial z} \quad (1)$$

where $f_0$ is the resonance frequency, $k$ is the spring constant, $F$ denotes the force between the tip and the sample, and $z$ is the tip–sample distance [App. Phys. Lett. 84, 10,8 (2004)]. This relationship remains physically valid from the attractive (non-contact) regime, through the onset of contact, and into the repulsive regime. The application of FM-AFM to the contact regime is well-established; for instance, Ternes *et al.* [Phys. Rev. Lett. 106, 016802 (2011)] successfully utilized $\Delta f$ measurements to analyze short-range chemical forces in metallic point contacts. In our experiments, measuring $\Delta f$ even in the $z < 0$ regime allows us to detect the magnetic exchange forces.

Following the reviewer's suggestion, we have inserted the following text in the manuscript

Page 2 of main text *"In this mode, the frequency shift ($\Delta f$) allows the determination of the force between the tip and the sample from the attractive regime to the repulsive regime (see also the Supporting Information) [App. Phys. Lett. 84, 10,8 (2004), Phys. Rev. Lett. 106, 016802 (2011)]."*

**Reviewer Comment 1.4** — - A questionable point to me is that the authors are sure to exclude the influence of the tunneling electrons on the switching in the $z$ regime ($< 0$) and bias voltage of 0.2 mV (page 2, lines from 60). In this point contact regime, quantum conductance should be considered (see Ref. [W. Paul et al. Nat. Phys. 13, 403–407 (2017)]). An in-depth discussion on this point can make the force-induced switching more convincing.

**Reply**: We appreciate the reviewer for raising this point. As shown in Fig. S7d, the conductance in the point contact regime ($z < 0$) deviates from exponential decay, exhibiting characteristics of quantum conductance similar to those reported by Paul et al. [Nat. Phys. 13, 403–407 (2017)]. However, it is important to note that the applied bias voltage in our experiment is limited to 200 $\mu$V. At this low bias, spin switching for $Ho_{top}$ is typically inhibited due to the high energy barrier between the $Ho_{up}$ and $Ho_{down}$. This suggests that the observed switching is not a result of tunneling current but involves strain-induced state mixing via the atomic probe.

Following this suggestion, we have added an in-depth discussion on the manuscript

Page 7 of the supporting information *"As shown in Fig. S7d, the conductance in the contact regime deviates from exponential decay, exhibiting characteristics of quantum conductance [W. Paul et al. Nat. Phys. 13, 403–407 (2017)]. However, the applied bias voltage is limited to 200 $\mu$V. Under these conditions, spin switching for $Ho_{top}$ is inhibited due to the energy barrier between the $Ho_{up}$ and $Ho_{down}$ as shown in Fig.1b. This suggests that the observed switching is not a result of tunneling current but involves strain-induced state mixing via the atomic probe."*

**Reviewer Comment 1.5** — - The authors briefly mentioned intra- and inter-atomic couplings at a contact regime as the origin of the crossover between FM and AFM tip-Ho interaction (page 3, lines 99-105). As the experimental $z$-trace data shown in Fig.3 clearly revealed the crossover, the authors doesn't have to rely on the previous works but can give a detailed discussion on the specific case in this work (Co tip and $Ho_{top}$ atom), which would strengthen the impact of the current work.

**Reply**:

We thank the reviewer for this constructive suggestion and for the positive assessment of our experimental data. We agree that the clear crossover observed in the $z$-trace (Fig. 3) allows for a specific discussion on the interaction between the Co tip and the Ho adatom, rather than relying solely on general references. To address this, we have expanded the discussion regarding the crossover from ferromagnetic to antiferromagnetic coupling.

Following the reviewer's suggestion, we have added the following sentence to the manuscript

Page 3 of the main text *"The transition from ferromagnetic to antiferromagnetic coupling is reminiscent of the Bethe–Slater curve. The antiferromagnetic coupling between the highly localized 4$f$ electrons in the Ho adatom and the 3$d$ electrons in the Co tip can be explained by an intra-atomic ferromagnetic coupling between the 4$f$ and 5$d$ (or 6$s$) electrons within the Ho adatom, and an inter-atomic antiferromagnetic coupling between the 5$d$ (or 6$s$) electrons of Ho and the 3$d$ electrons of the Co atom."*

**Reviewer Comment 1.6** — MINOR: - Superparamagnetic nature of a magnetic tip apex has been thoroughly studied in the works [Appl. Phys. Lett. 103, 032407 (2013)] and [Nano Convergence 4, 8 (2017)], which can be referred in the page 2, line 59.

**Reply**: We thank the reviewer for bringing these relevant works to our attention. Accordingly, we have included these references on the main text page 2 to support our discussion.

**Reviewer Comment 1.7** — - A clear example of "controlling the spin by manipulating the adsorption-site symmetry" (page 4, lines 158-159) has been recently published: [Nat. Commun. (2026), https://www.nature.com/articles/s41467-026-68314-6].

**Reply**: We are grateful to the reviewer for pointing out this very recent and relevant work. Accordingly, we have included this reference in the main text on page 4 and 5 to strengthen the context of our findings.

**Reviewer Comment 1.8** — - Fig.1b: A finite external magnetic field lifts the degeneracy of the two zero-field ground states (indicated by the red and blue arrows), and the authors try to show it in the figure, but it is not that visible in the energy scale of ∼300 meV, including the whole eigenstates of the system. How about using an inset just for the two states of the interest using an energy scale, comparable to the Zeeman energy splitting of the states? The central part of Fig.1b looks pretty empty.

**Reply**: We appreciate the reviewer's constructive suggestion regarding Fig. 1b. Accordingly, we have added an inset in the central part of Fig. 1b that zooms in on the lowest energy eigenstates.

---

# Reviewer 2

**Reviewer Comment 2.1** — Adachi et al. report force-based reading and writing of magnetic states of Ho adatoms on MgO using magnetic exchange force microscopy. The authors propose that mechanical manipulation offers a low-dissipation and highly localized alternative to conventional electrical reading and writing of single-atom magnets. Although the experiments are technically demanding and carefully carried out, it remains unclear whether the work constitutes a significant advance to justify publication in Nature Communications. In particular, the manuscript does not convincingly demonstrate that mechanical readout and writing provide a clear conceptual or practical advantage over electrical reading and writing schemes (as in Ref. 5).

**Reply**: We sincerely thank the reviewer for recognizing that our experiments are 'technically demanding and carefully carried out'. The most conceptual advantage of mechanical reading and writing is that it does not require a tunneling current. STM-based schemes (Ref. 5) are strictly limited to conducting substrates to flow the current. In contrast, our force-based method enable reading and writing of a single atom magnet on insulating substrates, such as bulk MgO.

According to the reviewer's comment, we have added the following sentence to the manuscript

Page 2 of the main text *"Since our proposed method enables the reading and writing of single-atom bits by force, it can be applied to various surfaces, including electrically insulating substrates that are inaccessible to conventional electrical techniques."*

**Reviewer Comment 2.2** — The force-based readout requires probing times of several seconds to tens of seconds due to the slow tip approach, whereas electrical readout based on

tunnel current in STM-based schemes can be performed on much shorter timescales. This slow operation may limit the practical relevance of the mechanical approach for memory applications. If the tip could approach very fast to reduce the readout time, it may induce spin transitions which disturbs the Ho state.

**Reply**: We clarify that the slow tip approach (vertical $z$-spectroscopy) described in the manuscript (for example Fig.3a) was performed specifically to quantify the force between tip and sample. Deriving the force $F$ requires integrating the frequency shift $\Delta f$ along the $z$-axis ($F \propto \int \Delta f dz$), which necessitates a slow and careful approach. However, for the practical purpose of reading $Ho_{up}$ and $Ho_{down}$, acquiring the full force curve is unnecessary. Instead, the readout can be performed by keeping the tip at a fixed height and monitoring the value of $\Delta f$, as shown, for example, in Fig. 4a. This fixed-height operation is analogous to the standard STM readout scheme, where the tunneling current is monitored at a constant tip height. In this mode, the readout speed is not limited by the $z$-piezo response but largely by the bandwidth of the frequency demodulation.

**Reviewer Comment 2.3** — The reading method also appears intrinsically invasive. The reading relies on strong tip-atom interactions, including strong magnetic fields from the Co-functionalized tip and the scattering electrons from the tip apex. As a result, the measured lifetime using forces (several seconds) is comparable to the current-based STM measurement.

**Reply**: As demonstrated in Fig. 5(a,b), we successfully distinguish between spin-up and spin-down states at a tip-sample distance of approximately 20 pm away from the point contact. In this near-contact regime, scattering electrons from the tip apex is suppressed. According to the reviewer comment, we have added the following sentence to the manuscript

Page 5 of the main text "In this near-contact regime, electron scattering from the tip apex is suppressed. Consequently, our force-based approach remains intrinsically non-invasive during readout."

Regarding the influence of the magnetic field from the Co-functionalized tip, we do not observe spontaneous spin reversal during spin readout. Therefore, we believe that the spin lifetime during readout remains long. The lifetime of several seconds mentioned by the reviewer was observed during the writing process. Although a potential concern could be Landau–Zener tunneling (LZT) induced by the stray field of the magnetic tip, we can confidently rule out LZT as the mechanism for spin reversal in our experimental setup, as discussed in detail in our response to Comment 3.2.

**Reviewer Comment 2.4** — While the authors argue that electrical currents lead to Joule heating, this effect on reading and writing atoms is typically negligible under standard STM operating conditions (from several hundred millikelvin to a few kelvin).

**Reply**: We believe that the cooling itself becomes a substantial challenge when current-driven operations are employed in high-density atomic memory arrays. In such systems, cumulative Joule heating can create a significant thermal load. Indeed, the critical need to mitigate such Joule heating is widely recognized, serving as a primary motivation for extensive research into multiferroic devices and electric-field-controlled spin manipulation. Therefore, we believe that avoiding

current-induced heating offers a distinct advantage for future device integration. According to the reviewer comment, we have added the following sentence to the manuscript

Page 1 of the main text *"Such heating becomes a critical issue in high-density atomic memory arrays, where the cumulative heat can be significant."*

**Reviewer Comment 2.5** — Several additional points would benefit from clarification: 1. The experiments were performed at very small tip-atom distances, close to or beyond the point-contact regime. Under these conditions, changes in the tip apex configuration are likely during readout. If so, it could make it difficult to reliably distinguish the two Ho spin states, as the frequency shift depends sensitively on the detailed tip configuration.

**Reply**: Figures 5(a) and 5(b) show frequency shift images obtained using the same tip. While Fig. 5(a) displays two Ho atoms in the spin-up state, Fig. 5(b) shows the state after one atom was switched to spin-down. If the tip apex configuration had changed during the operation, the imaging contrast for both Ho atoms would have changed simultaneously. However, as analyzed in Fig. 5(c), the contrast changed only for the manipulated atom, while the unswitched reference atom remained identical. This observation allows us to rule out the possibility of accidental tip change during spin manipulation. According to the reviewer comment, we have added the following sentence to the manuscript

Page 5 of the main text *"using the same tip in Fig.5a"*

Page 5 of the main text *"In Fig.5c, the contrast changed only for the spin switched Ho atom, while the unswitched reference Ho atom remained identical. This observation allows us to rule out the possibility of a tip change during the Ho spin manipulation."*

**Reviewer Comment 2.6** — 2. The claim that force-induced switching is "more spatially localized" because the switching rate decays more rapidly with distance (Fig. 4c) is not fully convincing. The measured decay mainly reflects the vertical dependence of the interaction. However, addressability in dense atomic arrays is determined by the lateral extent of the perturbation. A short vertical decay does not necessarily imply narrow lateral control, particularly for mechanically mediated interactions and magnetic stray fields.

**Reply**: We thank the reviewer for this important comment. We agree that the distinction between vertical decay and the lateral extent of the perturbation should be carefully considered. To ensure accuracy and avoid ambiguity, we have removed the following sentence from the manuscript:

Page 4 of the main text *"These results suggest that force-based spin switching enables a more spatially localized control of the spin orientation in the Ho adatom."*

**Reviewer Comment 2.7** — 3. The transition from ferromagnetic to antiferromagnetic coupling is mentioned but not clearly explained. Adding one or two brief sentences to outline the physical origin of this transition would improve clarity and help readers follow the discussion without having to consult the cited literature in detail.

**Reply**: We thank the reviewer for this helpful suggestion. We agree that providing a brief physical explanation for the observed transition significantly improves the clarity and self-sufficiency of the manuscript. Accordingly, we have inserted the following sentence to outline the mechanism.

Page 3 of the main text *"The transition from ferromagnetic to antiferromagnetic coupling is reminiscent of the Bethe–Slater curve. The antiferromagnetic coupling between the highly localized $4f$ electrons in the Ho adatom and the $3d$ electrons in the Co tip can be explained by an intra-atomic ferromagnetic coupling between the $4f$ and $5d$ (or $6s$) electrons within the Ho adatom, and an inter-atomic antiferromagnetic coupling between the $5d$ (or $6s$) electrons of Ho and the $3d$ electrons of the Co atom."*

---

# Reviewer 3

**Reviewer Comment 3.1** — The work "Force-Based Reading and Writing of Individual Single-Atom Magnets" by Adachi and coworkers reports on the timely investigation of the magnetic switching of single atom magnets using an atomic force microscope. The results are interesting because holmium single atom magnets on MgO have been shown to exhibit unusual magnetic bistability and their reading and writing have been largely based on current pulses using a scanning tunneling microscope and magnetic field sweeps.

**Reply**: We thank the reviewer for the positive assessment of our work and for recognizing the timeliness of our investigation into force-based switching of single-atom magnets.

**Reviewer Comment 3.2** — In their work, Adachi and coworkers claim that they can read and write these single atom magnets by force interaction with the tip of an atomic force microscope. Their rationale is that the Ho atoms in the MgO top site (residing above an O atom) have magnetic bistability but Ho adatoms on MgO bridge site exhibit mixed ground states. They claim that by bringing the tip close enough to the Ho atom, it slightly shifts position from top to bridge, essentially moving from the bistable to the mixed state and back which may result in a spin flip/state reversal.

While we follow and tend to agree with the reading part using non-contact force microscopy, showing nice frequency shift contrast between the Ho-up and Ho-down states, the current evidence does not yet substantiate force-based writing. Looking at previous reports on magnetic switching of Ho single atom magnets, it appears essential to rule out the magnetic field sweep via the hyperfine coupled levels as the cause for state reversals. Presently, Adachi and colleagues use magnetic tips and oscillations amplitudes of 65 pm that could randomize the states via Landau Zener tunneling, as the motion of a magnetic field is akin to performing magnetic field sweeps, as was previously shown.

**Reply**:

[Figure Redacted]

We thank the reviewer for raising this point regarding the mechanism of state reversal. Based on the energy diagram of the Ho/MgO system, we can exclude Landau-Zener tunneling (LZT) as the cause of the switching observed in our experiment. We illustrate this logic using Fig. R1.

1. **Absence of avoided level crossings at the operating field:** As shown in Fig. R1a, the avoided level crossings relevant to LZT (indicated by red circles and detailed in Fig. R1b) occur within a magnetic field range of approximately $\pm 200$ mT. Our writing experiments were performed under an external magnetic field of $B_{\text{ext}} = 3.0$ T (indicated by the blue dashed line in Fig. R1a). Therefore, our experiment was conducted far from the avoided level crossings.

2. **Influence of the tip's stray field:** Even considering the magnetic field generated by the tip ($B_{\text{tip}}$), our conclusion remains valid. Since the Co tip is magnetized by the external field ($3.0$ T), its magnetic moment is aligned parallel to $B_{\text{ext}}$. Therefore, the stray field from the tip *adds* to the external field. The total magnetic field experienced by the Ho atom is:

$$B_{\text{total}} = B_{\text{ext}} + B_{\text{tip}} > 3.0 \text{ T} \tag{2}$$

   Consequently, the oscillation of the tip (which modulates the tip-sample distance) changes the magnitude of $B_{\text{total}}$, but strictly maintains the value above 3.0 T (indicated by the blue arrow in Fig. R1a).

To clarify this point, we have added the following text:

Page 10 of the supporting information *"Additionally, we discuss the possibility that the oscillation of the magnetic tip could induce magnetic field sweeps across avoided level crossings, potentially leading to Landau–Zener tunneling (LZT) [Proc. R. Soc. London, Ser. A **137**, 696 (1932)]. Hyperfine interactions create avoided level crossings, typically within a magnetic field range of approximately ±200 mT [Phys. Rev. B **100**, 180405(R) (2019)]. Previous studies have demonstrated that, under zero external magnetic field, sweeping the magnetic field from the tip across these crossings can result in spin reversal [Phys. Rev. B **100**, 180405(R) (2019)]. However, in our experiment, we apply an external magnetic field of 3.0 T, which is far beyond the ±200 mT range. Furthermore, the magnetization of the Co tip aligns with the external magnetic field. Consequently, the stray field from the tip adds to the external field. Thus, we conclude that the observed spin switching is not driven by LZT."*

**Reviewer Comment 3.3** — The claimed force-based switching would accordingly require using truly nonmagnetic tips, performing the partial manipulation of the Ho atom from top towards the bridge site and then checking the Ho state using a magnetic read out, either via switching analysis, frequency shift, or ESR. State reversals may indeed occur in the authors' experiment; however, the present data do not isolate force-based switching from magnetic-field–induced transitions. In the light of these reservations and because force-based switching is prominently encoded in the title, we advise against publication in the present form until this major item is properly addressed. To substantiate force-based writing, the

authors must demonstrate reversible state control using a nonmagnetic tip, exclude magnetic-field–induced transitions via quantitative modeling of the tip field, and provide external-field and hyperfine-resolved switching statistics. We also mention here that substantial experimental evidence is relayed to the supplement. We believe that all major claims need to be sufficiently corroborated within the main part of the article already.

**Reply**:

We appreciate the reviewer's constructive criticism regarding the verification of the force-based switching mechanism. To perform the experiment suggested by the reviewer, we believe it would be necessary to first read the Ho spin state using a magnetic tip, then manipulate the Ho spin using a non-magnetic tip, and finally read the Ho spin state again with a magnetic tip. However, in our experiment setup, the magnetic tips are created by picking up approximately 20–30 Co atoms to maximize the stability of the magnetic tip. Reverting such a tip to a non-magnetic state by precisely redepositing the picked-up Co atoms was unsuccessful. While we recognize that methods like charge state manipulation [Nature 624, 64 (2023)] offer a promising route for non-magnetic tip experiments to readout the spin state of target single atom, we believe that implementing such a new experimental scheme lies outside the scope of the present work and is a subject for future study.

Regarding the request to exclude magnetic-field-induced transitions via quantitative modeling of the tip field, we have addressed this issue in detail in our Reviewer Comment 3.2.

We believe that the primary experimental data substantiating our major claims are contained within the main text. For example, the experimental results for the "reading" single atom magnet are presented in Fig. 3a, and the experimental results for the "writing" single atom magnet are shown in Fig. 4a.

**Reviewer Comment 3.4** — We also have smaller reservations listed below that would warrant revisions. Items in no particular order: -no justification given for the choice of 3T magnetic biasing field

**Reply**: We appreciate the reviewer's comment regarding the choice of the magnetic field. We selected $3.0\,\mathrm{T}$ to ensure the system is well outside the range of hyperfine-induced level crossings (typically within $\pm 200$ mT), thereby avoiding LZT. Additionally, our calculations (Fig. 1b, Fig. S3b, and Fig. S14(a-d)) confirm that the spin reversal mechanism is qualitatively consistent at $1.0\,\mathrm{T}$, $3.0\,\mathrm{T}$, and $8.0\,\mathrm{T}$, respectively. We have added a justification to clarify this rationale.

Experimental section *"to avoid Landau–Zener tunneling (LZT) (see also section 15)"*

Page 10 of the supporting information *"However, in our experiment, we apply an external magnetic field of 3.0 T, which is far beyond the ±200 mT range."*

Page 10 of the supporting information *"According to Fig.1b, Fig.S3b and Fig.S14(a-d), the spin reversal mechanism driven by the adsorption site change from top to bridge is qualitatively consistent at $1.0\,\mathrm{T}$, $3.0\,\mathrm{T}$, and $8.0\,\mathrm{T}$. Therefore, we choose $B = 3.0\,\mathrm{T}$ for the experimental measurements."*

**Reviewer Comment 3.5** — -Ho adatoms would not be stable at zero field without hyperfine interaction, which is not addressed by Adachi

**Reply**: We agree with the reviewer that the hyperfine interaction is critical for the stability of Ho adatoms at zero field. We have added the text to explicitly acknowledge this mechanism and included the relevant citation.

Page 2 of the main text *"Notably, spin switching at zero magnetic field is prevented by the hyperfine interaction [Phys. Rev. B **100**, 180405(R) (2019)]."*

**Reviewer Comment 3.6** — -oscillation amplitude only cited in figure captions and not in experimental section

**Reply**: Following the reviewer's suggestion, we have included the oscillation parameters in the Experimental Section to address this omission.

Page 2 of the main text *"with amplitude $A = 65\,\mathrm{p}m$ "*

**Reviewer Comment 3.7** — -electronic switching threshold around 73meV with transverse and of order and below 120meV without transverse magnetic field, so using 120mV to monitor Ho state could already lead to current induced state reversal

**Reply**: We agree with the reviewer that $120\,\mathrm{mV}$ is above the switching threshold. To clarify that the Ho spin is indeed in a switching regime at this voltage (before being stabilized at lower bias), we have inserted the following sentence into the manuscript

Page 2 of the main text *"At $V = 120\,\mathrm{mV}$, the Ho spin undergoes current-induced switching."*

**Reviewer Comment 3.8** — -Adachi and coworkers mention "exceeding threshold" for instance on p1L68 but do not clarify what parameters they are referring to.

**Reply**: We have added the word "distance" to the corresponding sentence in the text.

Page 3 and page 4 of the main text *"distance"*

**Reviewer Comment 3.9** — -Fig 4. Redundant panels (a) and (b)

**Reply**: We agree with the reviewer that there was redundancy between panels (a) and (b). We have removed the original Figure 4(a). Accordingly, the subsequent panels have been re-labeled (the original Fig. 4(b) is now Fig. 4(a), etc.), and the figure caption has been updated to reflect this change.

**Reviewer Comment 3.10** — -no mention of exchange and dipolar magnetic fields created by the cobalt functionalized tip

**Reply**: Regarding the exchange interaction, we have already clarified that it is not the primary driving force of the spin reversal. As stated in the main text (page 4), "Moreover, the observation of bidirectional spin switching rules out exchange forces as the driving mechanism for spin switching." As for the dipolar magnetic field, the stray field was explicitly included in our analysis (as detailed in our response to Comment 3.2), and we confirmed that it does not induce LZT.

**Reviewer Comment 3.11** — -missing switching histogram that should reveal state preference for Ho state aligned with external magnetic field

**Reply**: We have included the switching histogram as Fig.S10d to illustrate the state distribution. Accordingly, we have added the following sentence to the manuscript

Page 8 of the supporting information *"The histogram in Fig.S10d indicates a higher population of Ho↑, revealing a preference for the state aligned with the external magnetic field."*

**Reviewer Comment 3.12** — -no discussion of energetics Ho state aligned with magnetic field has a meV preference to the opposite state

**Reply**: We have included a discussion on the energetics of the Zeeman splitting to clarify the stability of the aligned state. Specifically, we added the following sentence to the manuscript

Page 2 of the main text *"In Fig.1(b), under an external magnetic field of 3.0 T, the Ho state aligned with the magnetic field has an energy preference of approximately* $3.5\,\mathrm{meV}$ *over the opposite state."*

**Reviewer Comment 3.13** — -Stevens parameters from SI differ from cited parameters for J=8

**Reply**: We derived the Stevens parameters ($B_n^k$) for both bridge and top sites using a point-charge model based on atomic configurations and charge states from the literature. Specifically, the positions of Ho, Mg, and O atoms were adopted from the structural data reported in the PhD thesis of T. Bilgeri [*Quantum dynamics in individual surface spins*, EPFL, 2021], while the charge state of the Ho adatom was set to $Q_{\mathrm{Ho}} = +3e$, following Natterer et al. [*Nature* **543**, 226–228 (2017)]. Although our parameters deviate slightly from those reported by Donati et al. [*Phys. Rev. Lett.* **121**, 027201 (2018)], presumably due to small differences in the modeled local coordination and charge state, our calculated energy diagrams exhibit similar qualitative behavior and magnetic field dependence.

**Reviewer Comment 3.14** — -discussion of Landau Zener tunneling missing

**Reply**: We have discussed the Landau-Zener tunneling in our response to Comment 3.2.

**Reviewer Comment 3.15** — -no dependence on external magnetic field shown

**Reply**: To address the dependence on the external magnetic field, we have added Figure S14 and the following text to the Supporting Information.

Page 9 of the supporting information *"Fig.S14a presents the energy diagram for* $\mathrm{Ho_{top}}$ *calculated at* $B = 1.0\,\mathrm{T}$*. The ground and metastable states are characterized by* $\langle J_z \rangle = -8.0$ *and* $\langle J_z \rangle = +8.0$*, respectively, with an energy splitting of* $1.16\,\mathrm{meV}$*. These states are dominated by the* $|m\rangle = \pm|8\rangle$ *states with more than* $99\,\%$ *weight. No* $\Delta m = \pm 1$ *or* $\Delta m = 0$ *transitions are allowed between Ho↑ and Ho↓. In contrast, Fig.S14b shows the energy-level diagram for* $\mathrm{Ho_{bridge}}$ *at* $B = 1.0\,\mathrm{T}$*. Here, the ground and metastable states exhibit* $\langle J_z \rangle = -5.5$ *and* $\langle J_z \rangle = +5.5$*. Notably, these states allow for a* $\Delta m = 0$ *transition with an energy difference of*

$1.2\,\mathrm{meV}$. *At* $B = 8.0\,\mathrm{T}$, *Fig.S14c shows the diagram for Ho*$_{\mathrm{top}}$. *The ground and metastable states are* $\langle J_z \rangle = -8.0$ *and* $\langle J_z \rangle = +8.0$ *with a larger energy splitting of* $9.2\,\mathrm{meV}$. *These states are dominated by the* $|m\rangle = \pm|8\rangle$ *states with more than* $99\,\%$ *weight. No* $\Delta m = \pm 1$ *or* $\Delta m = 0$ *transitions are allowed between Ho*$\uparrow$ *and Ho*$\downarrow$. *Figure S14d displays the energy diagram for Ho*$_{\mathrm{bridge}}$ *at* $B = 8.0\,\mathrm{T}$. *The ground and metastable states show* $\langle J_z \rangle = -6.8$ *and* $\langle J_z \rangle = +6.6$, *and they exhibit an allowed* $\Delta m = 0$ *transition with an energy difference of* $7.8\,\mathrm{meV}$. *According to Fig.1b, Fig.S3b and Fig.S14, the spin reversal mechanism driven by the adsorption site change from top to bridge is qualitatively consistent at* $1.0\,\mathrm{T}$, $3.0\,\mathrm{T}$, *and* $8.0\,\mathrm{T}$. *Therefore, we choose* $B = 3.0\,\mathrm{T}$ *for the experimental measurements."*

**Reviewer Comment 3.16** — -claims of higher spatial switching resolution not clear

**Reply**: We agree with the reviewer that the claim regarding higher spatial switching resolution is ambiguous. To ensure accuracy and avoid ambiguity, we have removed the following sentence from the manuscript:

Page 4 of the main text *"These results suggest that force-based spin switching enables a more spatially localized control of the spin orientation in the Ho adatom."*

**Reviewer Comment 3.17** — -role of hyperfine interaction

**Reply**: We have addressed the role of the hyperfine interaction in our response to Reviewer Comment 3.2 and 3.5. As detailed in Reviewer Comment 3.2 and 3.5, while the hyperfine interaction is crucial for the stability of the Ho spin near zero magnetic field, our experiments are conducted at $3.0\,\mathrm{T}$. Consequently, the hyperfine interaction plays a negligible role in our work.

---

## Reviewer 1

**Reviewer Comment 1.1** — The authors clarified point-by-point all the comments from me and revised the manuscript in appropriate ways. The revision is now in a good shape for publication in Nature Communications. I recommend publication of the manuscript as it is.

**Reply**: We thank the reviewer for their positive assessment of our revised manuscript and for supporting its publication in its current form.

---

## Reviewer 2

**Reviewer Comment 2.1** — The authors have addressed several of the previous comments, and the manuscript has improved. However, a few points would still benefit from further clarification.

1. The authors state that "we believe that the spin lifetime during readout remains long." Does Fig. 4a correspond to the reading or writing process? If it is a reading, the lifetime is only several seconds, which does not seem particularly long compared with the current-based readout scheme. For clarity, it would be useful to display the frequency-shift signals for both the readout and writing processes in the same figure.

**Reply**: We thank the referee for this question. Figure 4a corresponds to the writing process; therefore, the observed lifetime is only several seconds. We have added Fig. S15a and a new section to the Supporting Information (page 10), entitled "The Ho spin lifetime" along with the following sentences.

Figure S15a demonstrates the effect of the tip–sample distance on spin stability. In the initial stage ($0\,\mathrm{s} \leq t \leq 49\,\mathrm{s}$), the Co tip is positioned at a relatively large tip–sample distance. In this regime, no spin switching occurs within $49\,\mathrm{s}$. As the Co tip is brought closer to the sample

($t > 49$ s), the spin lifetime is reduced to several seconds, as evidenced by the random telegraph signal.

**Reviewer Comment 2.2** — In addition, what is the longest spin lifetime that can be achieved using the force-based readout method?

**Reply**: By exponentially extrapolating the data in Fig. 4b, we estimate that the spin lifetime exceeds $1000\,\mathrm{s}$ at $z = 20\,\mathrm{pm}$. Experimentally, we have confirmed a stable readout for up to $218\,\mathrm{s}$ based on Fig. 5a and 5b. Accordingly, we have added the following sentence to the revised manuscript:

"In Figs.5a and 5b, based on the time required to image a single Ho, the Ho spin can be stably read out for at least $218\,\mathrm{s}$."

**Reviewer Comment 2.3** — 2. The statement that "In this near-contact regime, electron scattering from the tip apex is suppressed." may be incorrect. The scattering should increase exponentially with decreasing tip-atom distance [Nat. Phys. 13, 403 (2017)]]. Therefore, electron scattering in the near-contact regime would be expected to be significantly stronger than in the current-based readout scheme used in Ref. 5. In this sense, the proposed readout method may also be intrinsically invasive. It would be helpful if the authors could further clarify this point.

**Reply**: As the reviewer pointed out, our use of the term "suppressed" may be inappropriate. What we intended to convey is that, although electron scattering from the tip occurs, no $\Delta m = \pm 1$ or $\Delta m = 0$ transitions are allowed between Ho↑ and Ho↓ for $\mathrm{Ho_{top}}$. Therefore, spin reversal does not take place.

To avoid misunderstanding, we have deleted the following statement: "In this near-contact regime, electron scattering from the tip apex is suppressed.".

---

# Reviewer 3

**Reviewer Comment 3.1** — The authors have attempted to reply to the comments raised by the reviewers. While some have been satisfactory, we remain not convinced by the response to the major items and request further clarification.

The main issue is the unsatisfactory discussion of Landau Zener tunneling as a potential switching mechanism in their AFM investigation of the Ho single atom magnets. The statement that 3 T is far from the hyperfine levels is misleading. While it may be true that 3 T is large enough stabilize the Ho adatom; this holds true only in absence of the tip or when the tip-sample separation is large. However, the supposed force based switching requires a close proximity of the tip-apex with the Ho adatom and the Ho adatom therefore experiences a different effective field than it that simple picture. The authors must prove and rule out that the essential point contact between Co and Ho does not cause a different scenario. This

close separation of a magnetic tip with a magnetic adatom is the regime where exchange forces dominate and have been shown to easily exceed 3 T, for instance Yang, Phys. Rev. Lett. 122, 227203 (2019).

**Reply**:

*[Redacted]*

We thank the reviewer for raising this important point regarding the effective magnetic field at close tip–sample separations. We agree that, as the magnetic tip approaches the target atom, the effective magnetic field experienced by the adatom increases. Importantly, in our experimental setup, the Co tip behaves as a superparamagnetic tip. Under an external magnetic field of $3.0\,\mathrm{T}$, the magnetization of the tip aligns with the applied field. Consequently, as the Co tip approaches the Ho adatom, it further enhances the effective magnetic field beyond the external field ($> 3.0$ T). As shown in Fig. R1a, this implies that the effective magnetic field ($B_{\text{eff}}$) experienced by the Ho atom increases strictly beyond $3.0\,\mathrm{T}$. Because the effective field increases monotonically as the tip approaches, which is fully consistent with the observations for the Ti atom reported by Yang et al., the Ho atom is driven further away from the near-zero-field regime ($-200$ mT $< B <$ $+200$ mT) required for Landau–Zener tunneling (LZT) to occur. Therefore, we can rule out LZT as the switching mechanism under an external magnetic field of $3.0\,\mathrm{T}$.

We note that this point has been discussed in detail in the Supporting Information as follows:

Additionally, we discuss the possibility that the oscillation of the magnetic tip could induce magnetic field sweeps across avoided level crossings, potentially leading to LZT [Series A, Containing Papers of a Mathematical and Physical Character 137(833), 696–702 (1932)]. Hyperfine interactions create avoided level crossings, typically within a magnetic field range of approximately ±200 mT [Phys. Rev. B 100, 180405(R) (2019)]. Previous studies have demonstrated that, under zero external magnetic field, sweeping the magnetic field from the tip across these crossings can result in spin reversal [Phys. Rev. B 100, 180405(R) (2019)]. However, in our experiment, we apply an external magnetic field of 3.0 T, which is far beyond the ±200 mT range. Furthermore, the magnetization of the Co tip aligns with the external magnetic field. Consequently, the stray field from the tip adds to the external field [Phys. Rev. Lett. 122, 227203 (2019)]. Thus, we conclude that the observed spin switching is not driven by LZT.

For clarity, we have also added Reference [Phys. Rev. Lett. 122, 227203 (2019)] to the Supporting Information.

"Consequently, the stray field from the tip adds to the external field [Phys. Rev. Lett. 122, 227203 (2019)]]".

**Reviewer Comment 3.2** — We have already suggested a potential experimental verification that the authors dismissed. Here is another one: The authors could repeat the experiment at zero field, where Ho adatoms are stable and attempt to reverse the state at close proximity, where the exchange fields created by Co tip may now permanently move the Ho state towards a stable configuration for the writing period and the resulting state reversal would then be more closely related to force rather than to LZT. Alternatively, repeat the experiment at even more extreme fields and investigate whether the switching dynamics is field dependent.

**Reply**: Regarding the suggestion to perform the experiment at zero external magnetic field ($0\,\mathrm{T}$), we first note that the Co tip utilized in our study behaves as a superparamagnetic tip. In the absence of an external magnetic field, the magnetization of the tip fluctuates and cannot be stabilized in a unique direction. Consequently, readout of the spin state of the Ho adatom at $0\,\mathrm{T}$ is not possible. One possible approach is to use an antiferromagnetic tip at $0\,\mathrm{T}$, which would require the development of a method to locally attach a Cr tip to the length-extension resonator. We would like to consider this an important direction for future investigation.

Following the reviewer's comment, we conducted additional experiments at extreme magnetic fields ($8.0\,\mathrm{T}$) and included the results as Fig. S14(e–g). As shown in Fig. R1b, at $8.0\,\mathrm{T}$, the system is sufficiently far from the regime where LZT occurs, and thus behavior similar to that at $3.0\,\mathrm{T}$ is expected (the resulting state reversal more closely related to force than to LZT). Consistent with this expectation, we indeed obtained results comparable to those at $3.0\,\mathrm{T}$. We have also added the following description to the Supporting Information:

"Figure S14(e–g) presents the observation of Ho spin switching at $8.0\,\mathrm{T}$. As shown in the time-dependent $\Delta f$ traces (Figs.S14e and f), clear spin switching is observed with a spin lifetime on the order of several seconds, similar to the behavior at $3.0\,\mathrm{T}$. The tip–sample distance required to induce this switching is determined from the $\Delta f$ curve in Fig.S14g, which reveals that the switching occurs when the tip–sample distance is reduced to a very close regime ($z \leq$

$-143$ pm). Furthermore, the overall shape of the $\Delta f$ curve in Fig.S14g is qualitatively similar to that shown in Fig.S8a. These findings are fully consistent with the results discussed in the main text, suggesting that the Ho spin switching at $8.0\,\mathrm{T}$ is governed by the same underlying mechanism as at $3.0\,\mathrm{T}$—namely, a mechanism driven by a change in the adsorption site from a top site to a bridge site."

**Reviewer Comment 3.3** — One of the additional items mentioned by the authors is that the suggested force based magnetic switching could be applied to nonconducting surfaces. While it may be true, this is not necessarily a unique feature of a supposed force based reversal as the field-sweep technique introduced by Forrester Phys. Rev. B 100, 180405(R) (2019) already covers non conducting substrates. The latter field-sweep is incidentally noninvasive and can be made selective with appropriately sharp field gradients.

Unless these issues are properly addressed, we refrain from providing support for publication.

**Reply**: We thank the reviewer for this important comment and for pointing out the work by Forrester *et al.* [Phys. Rev. B 100, 180405(R) (2019)], which we have now cited in the revised manuscript. We agree that the field-sweep technique provides a noninvasive approach that can be applied to nonconducting substrates for writing the Ho spin state. That said, we would like to emphasize that our force-based approach enables both the reading and writing of single-atom spin states without relying on electrical current. In this sense, it offers a complementary route that is not limited by electrical accessibility and can, in principle, be extended to a wide range of surfaces, including bulk insulating substrates.

Accordingly, we have revised the manuscript as follows: "Since our proposed method enables the reading and writing of single-atom bits by force, it can be applied to various surfaces, including bulk insulating substrates that are inaccessible to conventional electrical techniques [Phys. Rev. B 100, 180405(R) (2019)]."

## Reviewer 2

**Reviewer Comment 2.1** — The authors have addressed my comments. I recommend publication. I have read the review and reply, and here is my comment.

As pointed out by Reviewer 3, the oscillating magnetic field generated by the tip could in principle induce Ho switching under an external magnetic field. The authors rule out this possibility by measuring the Ho switching at a higher external field (8 T), where they observe behavior similar to that at lower magnetic fields.

However, at such a short tip-atom distance, the tip field arising from exchange interaction is expected to dominate over the dipolar stray field. Therefore, the statement in the SI, "Furthermore, the magnetization of the Co tip aligns with the external magnetic field. Consequently, the stray field from the tip adds to the external field [27]," may not be fully accurate, as it appears to refer mainly to the dipolar contribution. I suggest the authors also discuss the role of oscillating exchange field in the Ho switching process.

**Reply**: We thank the reviewer for their positive assessment of our revised manuscript and for supporting its publication.

In our previous response, we used the term "the stray field" to refer to the exchange field; however, acknowledging that this could be misleading, we have decided to use the term "the exchange field" directly. Accordingly, we have updated the Supporting Information on page 11 to reflect this change.

We have ruled out an oscillating exchange field as the driving mechanism by measuring Ho switching at a higher external field (8T), where the observed behavior is similar to that at lower magnetic fields as we discussed in detail in our previous response to Reviewer 3. Moreover, as

stated on page 4 of the main text ("the observation of the bidirectional spin switching rules out exchange forces as the driving mechanism for spin switching"), we found that the switching mechanism in our study is not driven by exchange forces. Consequently, the oscillating exchange field is not the underlying mechanism for the observed switching in our work.
